# Poly-GP accumulation due to C9orf72 loss of function induces motor neuron apoptosis through autophagy and mitophagy defects

**DOI:** 10.1080/15548627.2024.2358736

**Published:** 2024-09-24

**Authors:** Hortense de Calbiac, Solène Renault, Grégoire Haouy, Vincent Jung, Kevin Roger, Qihui Zhou, Maria-Letizia Campanari, Loïc Chentout, Doris Lou Demy, Anca Marian, Nicolas Goudin, Dieter Edbauer, Chiara Guerrera, Sorana Ciura, Edor Kabashi

**Affiliations:** a Imagine Institute, INSERM UMR 1163, Team Translational Research for Neurological Diseases, Paris Descartes University, Paris, France; b Proteomics Platform 3P5Necker, INSERM US24/CNRS UMS, Paris Descartes University, Structure Fédérative de Recherche Necker, Paris, France; c German Center for Neurodegenerative Diseases (DZNE), Munich, Germany; d Munich Cluster of Systems Neurology (Synergy), Munich, Germany; e Imaging Core Facility, INSERM US24/CNRS UMS3633, Paris, France; f Ludwig-Maximilians-Universität (LMU) Munich, Graduate School of Systemic Neurosciences (GSN), Munich, Germany

**Keywords:** Amyotrophic lateral sclerosis, apoptosis, mitochondria, motor neuron, neurodegeneration, poly-GP

## Abstract

The GGGGCC hexanucleotide repeat expansion (HRE) of the *C9orf72* gene is the most frequent cause of amyotrophic lateral sclerosis (ALS), a devastative neurodegenerative disease characterized by motor neuron degeneration. *C9orf72* HRE is associated with lowered levels of C9orf72 expression and its translation results in the production of dipeptide-repeats (DPRs). To recapitulate *C9orf72*-related ALS disease *in vivo*, we developed a zebrafish model where we expressed glycine-proline (GP) DPR in a *c9orf72* knockdown context. We report that *C9orf72* gain- and loss-of-function properties act synergistically to induce motor neuron degeneration and paralysis with poly(GP) accumulating preferentially within motor neurons along with Sqstm1/p62 aggregation indicating macroautophagy/autophagy deficits. Poly(GP) levels were shown to accumulate upon *c9orf72* downregulation and were comparable to levels assessed in autopsy samples of patients carrying C9orf72 HRE. Chemical boosting of autophagy using rapamycin or apilimod, is able to rescue motor deficits. Proteomics analysis of zebrafish-purified motor neurons unravels mitochondria dysfunction confirmed through a comparative analysis of previously published *C9orf72* iPSC-derived motor neurons. Consistently, 3D-reconstructions of motor neuron demonstrate that poly(GP) aggregates colocalize to mitochondria, thus inducing their elongation and swelling and the failure of their processing by mitophagy, with mitophagy activation through urolithin A preventing locomotor deficits. Finally, we report apoptotic-related increased amounts of cleaved Casp3 (caspase 3, apoptosis-related cysteine peptidase) and rescue of motor neuron degeneration by constitutive inhibition of Casp9 or treatment with decylubiquinone. Here we provide evidence of key pathogenic steps in C9ALS-FTD that can be targeted through pharmacological avenues, thus raising new therapeutic perspectives for ALS patients.

## Introduction

A GGGGCC hexanucleotide repeat expansion (HRE) within the non-coding region of the *C9orf72* (C9orf72-SMCR8 complex subunit) gene is the most common known cause of familial and sporadic cases of amyotrophic lateral sclerosis (ALS), a devastating neurodegenerative disorder characterized by the loss of cortical and spinal motor neurons [1–4]. Neuropathological studies have shown that the HRE is translated into five distinct dipeptides repeats (DPRs) depending on the reading frame and direction of transcription: poly-GA, poly-GP, poly-GR are encoded by the sense strand, while poly-PA, poly-PR and poly-GP by the anti-sense strand [5–7]. Detectable levels of poly-GP in the cerebrospinal fluid/CSF have been reported in the pre-symptomatic phase of *C9orf72* mutation carriers [8] making it a candidate biomarker for *C9orf72*-ALS [8–12]. Indeed, the cerebrospinal fluid level of poly-GP was used as the read-out for the effectiveness of antisense therapy in both mouse models [13,14] and recently, in patients [15]. Despite its promising potential as a key biomarker of future therapies, the pathological consequence of ectopic poly-GP expression is not well established. In experimental models, overexpression of poly-GP by itself carries little to no toxicity [16–18], but an appropriate genetic context contributing to toxicity and eventual neurodegeneration could be lacking. Indeed, numerous reports, including ours, document the reduction of C9orf72 expression in heterozygous carriers of the HRE mutation [19–25], possibly due to hypermethylation and transcriptional downregulation of the gene [26–28]. Loss of C9orf72 have been associated with defects in autophagy at different steps of the process, from the regulation of its initiation to the lysosomal degradation [29–34]. Importantly, inhibition of autophagy has been shown to trigger the transition from soluble to insoluble DPRs [35] and leads to increased neuronal toxicity in cellular and animal models of C9orf72-ALS [36–39]. Indeed, in ALS pathological tissue, DPRs, represented mainly by poly-GP and poly-GA, accumulate in aggregates that are also immunopositive for the autophagy receptor SQSTM1/p62; indicating autophagy deficits [5,6,40].

To elucidate pathogenic mechanisms due to the C9orf72 mutation leading to neurodegeneration in ALS, we developed a zebrafish model where we combined both gain – and loss-of-function properties of *C9orf72* mutation. We report that *C9orf72* knockdown and poly(GP) expression act synergistically to induce partial paralysis resulting from motor neuron degeneration. We confirmed that motor neuron loss is due to autophagy deficiency and that chemical boosting of this pathway, is able to reduce poly(GP) and Sqstm1/p62 accumulation and to rescue motor neuron survival and zebrafish motor deficits. Importantly, we observed that, under *c9orf72* knockdown, poly(GP) accumulates within swollen and elongated mitochondria in motor neurons, which is associated to mitophagy defects. Consistent with these findings, treatment of zebrafish larvae with urolithin A, a mitophagy activator, improves zebrafish locomotion. Proteomics analysis of purified motor neurons confirms aberrant signaling of mitochondria; thus giving rise to the engagement of caspase cascade, which can be prevented with decylubiquinone (dUb) treatment of zebrafish larvae, or by the genetic restriction of Casp9 in motor neurons. In this study, we advance our understanding of the *C9orf72* mutation by describing a novel vertebrate model and the cascade of events leading to motor neuron loss, and point out four pharmacological compounds that target these pathways to counteract motor deficits by preventing neurodegeneration.

## Results

### Synergistic properties of C9orf72 mutation induce motor neuron degeneration and paralysis in vivo

**Figure 1.**
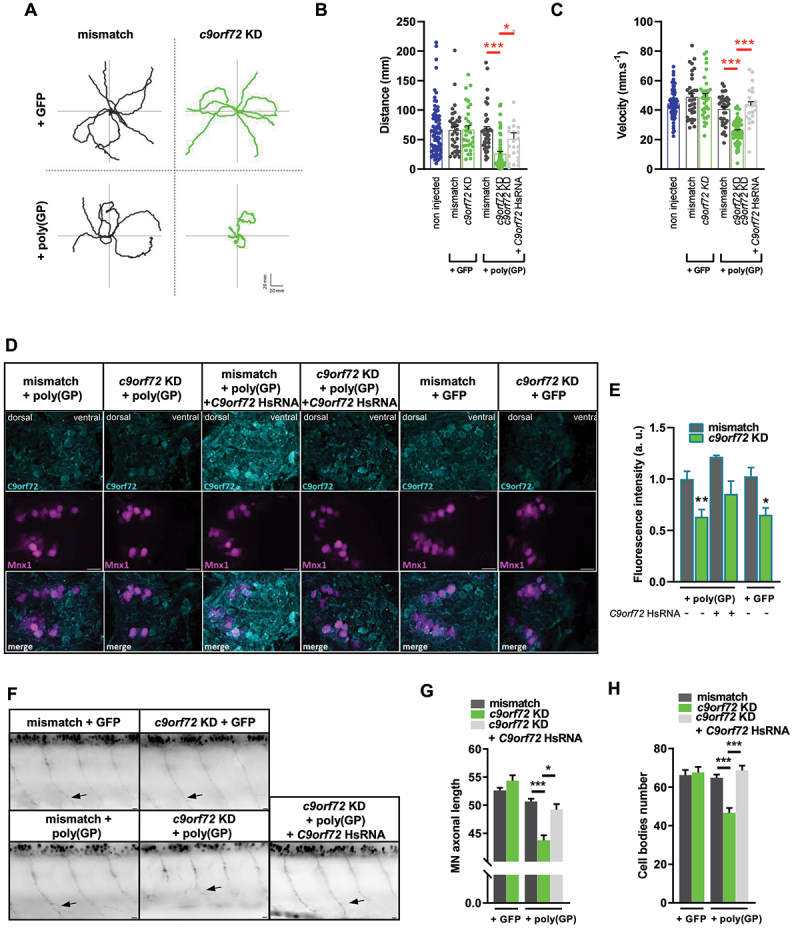
*C9orf72* knockdown is essential to trigger poly(GP) toxicity, inducing paralysis due to motor neuron degeneration.

Note: (**A**) Representative swimming trajectories of 50 hpf zebrafish showing locomotor defects in the *c9orf72* KD + poly(GP) condition only. (**B, C**) Quantification of average swimming distance (**B**) and velocity (**C**), reflecting the motor phenotype of 50 hpf zebrafish of the *c9orf72* KD + poly(GP) condition and (**B, C**) the capacity of *C9orf72* human RNA (HsRNA) to rescue these parameters. Each dot represents one embryo. (**D, E**) Representative images (**D**) and quantification of average fluorescence intensity (**E**) from C9orf72 immunolabeling in spinal sections of *Tg(mnx1:gal4)/(UAS:RFP)* 50 hpf zebrafish. Scale bar: 10 µm. (**F**) Representatives images of MN of *Tg(mnx1:gal4)/(UAS:RFP)* 50 hpf larvae illustrating the disruption of MN axonal beam and a lower density of MN cell bodies in *c9orf72* KD + poly(GP) condition. Arrows indicate an example of endpoint used for axonal length measurement. Scale bar: 10 µm. (**G**) Quantification of average MN axonal length to dorsal thickness ratio of 50 hpf zebrafish. *C9orf72* HsRNA rescues the length of the projections. (**H**) Quantification of average MN cell bodies number per 5 somites in 50 hpf zebrafish. *C9orf72* HsRNA rescues the density of spinal MN. * p < 0.05; ** p < 0.01; *** *p *< 0.001. Data are presented as mean + standard error of the mean (SEM).

To recapitulate the *C9orf72*-related aspects of ALS pathology *in vivo*, we generated a zebrafish model combining both the gain – and loss-of-function aspects of the *C9orf72* mutation simultaneously. Given that *C9orf72* mutation leads to reduced C9orf72 expression and haploinsufficiency [19–21,24,25], we knocked down the *c9orf72* zebrafish ortholog (*zgc:100846/*ENSDARG00000011837) using a specific antisense morpholino oligonucleotide (AMO) in order to implement a partial inhibition of its expression. We have previously established a “subphenotypic” dose for this AMO that leads to a reduction of *zgc:100846/C9orf72* expression [29]. The resulting partial knockdown (KD) of *zgc:100846/C9orf72* is not triggering a detectable motor phenotype nor morphological deficits in zebrafish embryos [29] as shown in Figure 1A and Fig. S1A with reduced c9orf72 levels measured in spinal cord sections upon the c9orf72 AMO injection as compared to control conditions (Figure 1D, E). Along with *c9orf72* KD, we overexpressed a construct encoding 51 repeats of poly(GP) fused to an N-terminal GFP tag, or expressed GFP alone as an internal control.

At 50 hpf, zebrafish embryos have stereotyped escape responses to touch, allowing for an assessment of motor ability using the Touch-Evoked Escape Response (TEER) test, as previously described [25,41,42]. Individual swimming episodes were traced for zebrafish co-expressing poly(GP) or GFP-only control plasmid together with the *c9orf72* AMO or its mismatch control (Figure 1A). Partial paralysis was observed in the *c9orf72* KD + poly(GP) condition, but not in the mismatch + poly(GP) and the *c9orf72* KD + GFP conditions, as shown by the swimming trajectories in Figure 1A. Indeed, quantitative analysis of the TEER demonstrated that expression of poly(GP) alone does not trigger any swimming abnormalities, as swimming distance and velocity of mismatch + poly(GP) embryos was not different from either non-injected or GFP control conditions (Figure 1B, C). However, concomitant downregulation of *c9orf72* and expression of poly(GP) induced a significant and specific reduction in motor abilities, as quantified by the reduced total distance (Figure 1B) and velocity (Figure 1C) of 50 hpf embryos, when compared to respective mismatch and GFP controls. To confirm the specificity of these motor phenotypic features, we performed functional rescue experiments using co-expression of human *C9orf72* long transcript mRNA (ENST00000380003.8, *C9orf72* HsRNA). Co-injection of *C9orf72* HsRNA at the one-cell stage was able to restore the swimming parameters of *c9orf72* KD + poly(GP) embryos up to control levels, as shown for total distance (Figure 1B) and velocity (Figure 1C) of swimming episodes. Importantly, the percentage of zebrafish larvae with motor deficits was substantially reduced upon co-expression of *C9orf72* HsRNA (Fig. S1A), confirming the functional contribution of c9orf72 lower expression to the motor phenotype. To ascertain that C9orf72 antisense oligonucleotides lead to c9orf72 downregulation, we show reduced immunolabeling of c9orf72 in transversal spinal cord sections using a specific C9orf72 antibody [36] in *c9orf72* KD + poly(GP) and *c9orf72* KD + GFP as compared to other control conditions (Figure 1D, E). Importantly, rescue of C9orf72 expression is observed upon co-expression of human *C9orf72* RNA (Figure 1D, E). These spinal cord sections were performed in double transgenic *Tg(mnx1:gal4)*/*Tg(UAS:RFP)* 50 hpf zebrafish, that display RFP fluorescence in both primary and secondary spinal motor neurons (Fig. S1B).

To assess whether the reduced locomotor capacity of *c9orf72* KD embryos expressing poly(GP) is related to any motor neuron defects, axonal projections from spinal motor neurons were measured at 50 hpf in *Tg(mnx1:gal4)*/*Tg(UAS:RFP)* 50 hpf zebrafish, when both primary and secondary motor neurons have reached their final targets, which revealed the combined toxicity of c9orf72 loss and poly(GP) expression on the axonal growth (Figure 1F), as quantified by the axonal length normalized to the dorsal thickness (Figure 1G); the latter remaining unaltered between all conditions (Fig. S1C). To define whether motor neuron degeneration was occurring in this model, we quantified the number of primary and secondary motor neurons in the fifteenth to the nineteenth intersomitic segments of 50 hpf *Tg(mnx1:gal4)/Tg(UAS:RFP)* embryos. A significant decrease of neuronal cell bodies was observed in the *c9orf72* KD + poly(GP) condition as compared to mismatch and GFP control conditions (Figure 1F, H), suggesting that the toxic effect of the combined gain and loss of function affects the survival of motor neurons. As described above for the swimming parameters and phenotype distribution, overexpression of *C9orf72* HsRNA is able to rescue both axonal length and survival of motor neurons (Figure 1F-H). Overall, these results demonstrate that gain and loss of function aspects of *C9orf72* HRE synergize to induce paralysis due to motor neuron degeneration.

We monitored the ventral axiogenesis of motor neurons by time lapse imaging of the intersomitic segments 15–18 from 19 hpf to 33 hpf, thus comprising the timeline for the complete axonal projections of the spinal primary motor neurons. We observed that the combination of *c9orf72* KD and poly(GP) expression induces an axonopathy of motor neurons as shown by shortened axonal projections in this condition when compared to mismatch + poly(GP) control, while axogenesis initiates at the correct time in both conditions (Fig. S1D).

To verify that the different phenotypes associated with poly(GP) expression (amino acid sequence provided in Figure S2A) are not an artifact from its C-term tag neither than from its GFP fusion, we used two other poly(GP) construct: poly(GP)_noTAG_ and poly(GP)_noGFP_, respectively. Our results show that in the *c9orf72* KD background the use of these variants of poly(GP) constructs induce a similar locomotor phenotype, as shown by TEER results in Fig. S1E and Fig. S2B, C, and induction of axonopathy and motor neuron loss (Fig. S1F), indicating that the fusion with GFP and the construct tags do not mediate its toxic effect.

### C9orf72 depletion induces poly(GP) and Sqstm1/p62 accumulation particularly in motor neurons

**Figure 2.**
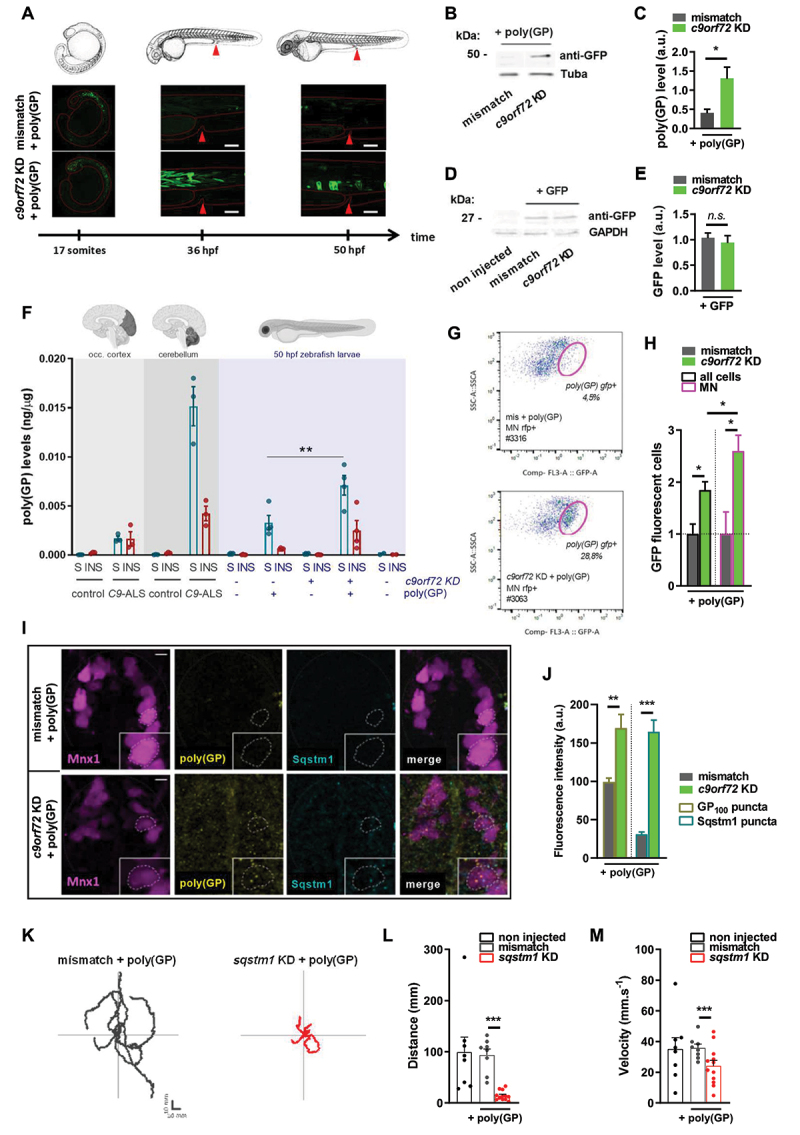
Poly(GP) and Sqstm1/p62 accumulate preferentially in motor neurons under *c9orf72* knockdown.

Note: (**A**) Representative images of poly(GP) expression through monitoring of GFP fluorescence in whole zebrafish at different time points: 17 somites, 36 hpf and 50 hpf, showing the accumulation of poly(GP) under *c9orf72* knockdown. The red arrowhead indicates the same location, between somites 14–15, in the schematic and in the fluorescent micrographs. Scale bar: 100 µm (**B, C**) Immunoblot (**B**) of poly(GP) at the expected size of 46 kDa using an anti-GFP antibody and quantification (**C**) of average poly(GP) peptide levels relative to Tuba/α-tubulin, showing increased levels of poly(GP) in *c9orf72* KD + poly(GP) condition (50 hpf). (**D, E**) Immunoblot (**D**) of GFP control expression at the expected size of 27 kDa and quantification (**E**) of average GFP protein levels relative to GAPDH, showing no difference between mismatch + GFP and *c9orf72* KD + GFP conditions (50 hpf). (**F**) Raw poly(GP) levels normalized to total input protein in soluble and insoluble fractions of control and *C9orf72* patients occipital cortex and cerebellum (gray rectangles, N = 3) compared to 50 hpf zebrafish (blue rectangle, N = 4) as detected by immunoassay. Controls for zebrafish expressing GFP-poly(GP) are zebrafish expressing GFP only (“poly(GP)”). (**G, H**) Representative GFP fluorescence distributions (**G**) in RFP-positive cells *i.e*. motor neurons from dissociated *Tg(mnx1:gal4)/(UAS:RFP)* 50 hpf zebrafish using flow cytometry and quantification (**H**) of poly(GP) positive cells, relative to all cells or to motor neuron population, showing general and motor neuronal accumulation of poly(GP) in *c9orf72* KD + poly(GP) condition. (**I, J**) Representative confocal images (**I**) and quantification of average fluorescence intensity (**J**) from poly(GP) and Sqstm1/p62 immunolabeling in spinal sections of *Tg(mnx1:gal4)/(UAS:RFP)* 50 hpf zebrafish, showing increased fluorescence intensity of poly(GP) and SQSTM1/p62 signals in *c9orf72* KD + poly(GP) condition. The dot lines in **I** encircle a representative motor neuron. A zoomed-in view is provided in the insert. Scale bar: 5 µm. (**K-M)** TEER results of 50 hpf zebrafish expressing poly(GP) with mismatch AMO, or in combination with the KD of *sqstm1*. Representations of swimming trajectories (**K**) of 50 hpf zebrafish resulting from the TEER test, and average swimming distance (**L**) and velocity (**M**), showing locomotor defects in *sqstm1* KD + poly(GP) condition. * p < 0.05; ** p < 0.01; *** p < 0.001 All data are presented as mean + SEM.

We monitored the expression of the poly(GP) repeats in our model by live imaging to determine if it is prone to accumulation. A few hours after the injection, at 17 somites, poly(GP) expression, as detected by the GFP fluorescence, was ubiquitous and equivalent between the control and *c9orf72* KD conditions (Figure 2A). However, we observed that poly(GP) is rapidly cleared over time in control condition, with little fluorescence observable at 50 hpf. In contrast, in the *c9orf72* KD condition, this clearance appears to be suppressed, as shown by the accumulated fluorescent signal in this condition (Figure 2A). To confirm these observations, we performed immunoblotting on protein extracts from 50 hpf embryos, using an anti-GFP antibody, which corroborate that poly(GP) levels is increased under *c9orf72* depletion (Figure 2B, C, Fig. S3A) as opposed to earlier timepoints in zebrafish embryonic development where poly(GP) labeling was similar in *c9orf72* KD and mismatch control conditions as shown in Figure 2A (17 somite panel). Poly(GP) was detected by the anti-GFP antibody at the correct size and we also confirmed its accumulation using a specific anti-poly(GP) antibody (“C9-RANT”, Fig. S3A, B). As a proof of principle, we also expressed a shorter number of GP repeats (10X) and validated that it is detected at the expected size (Fig. S3A). Importantly, at the earlier stage of 20 hpf, poly(GP) levels are similar between control and *c9orf72* KD conditions (Fig. S3C, D). At 50 hpf no difference in GFP protein levels was observed in the *c9orf72* KD + GFP only condition, as compared with the mismatch condition (Figure 2C, D), thus suggesting a specific role for c9orf72 in the clearance of poly(GP). Furthermore, we tested the effect of *c9orf72* KD on the persistence of a construct encoding an 89 repeat poly(GR) peptide chain (sequence in Fig. S2A) expressed at the same low concentration as poly(GP) in zebrafish, and we observed that *c9orf72* depletion does not have any effect on poly(GR) levels at 50 hpf as detected by Western blotting (Fig. S3E, F). Interestingly, locomotion of *c9orf72* KD + poly(GR) 50 hpf zebrafish was normal as compared to mismatch + poly(GR) zebrafish and GFP controls, as shown by average traveled travelled distance from TEER quantification (Fig. S3G). Overall, these results demonstrate that in our experimental conditions of low DPR expression, *c9orf72* KD induces the accumulation of poly(GP) preferentially, leading to a locomotor phenotype.

To determine if the expression of poly(GP) in our model was comparable to the detected GP levels in affected autopsy tissue obtained from ALS-FTD patients, we examined poly(GP) levels using an established immunoassay in 50 hpf zebrafish (Figure 2F). We observed that poly(GP) are enriched in the soluble fraction of total proteins (Figure 2F), as previously reported [38,43,44] and are remarkably comparable to the GP levels in zebrafish samples. Importantly, poly(GP) levels are significantly higher in zebrafish under *c9orf72* KD, as compared to the mismatch control (Figure 2F). Increased GP levels are measured in the soluble fraction of the cerebellum and the occipital cortex of patients carrying *C9orf72* mutation as compared to controls (Figure 2F). Furthermore, immunoassay analysis showed that GP was detected and increased in both soluble and insoluble fractions of the zebrafish samples when poly(GP) was combined with *c9orf72* KD as compared to controls. However, the GP increase in the insoluble fraction did not reach statistical significance (Figure 2F). These results are consistent with previous studies showing the majority of GP is found in the soluble fraction [38,43,44].

Given that motor neuron loss occurs under the synergistic toxicity of *c9orf72* loss and gain of function (Figure 1F-H), we examined whether poly(GP) preferentially accumulates in motor neurons in regards to other cellular populations. To answer this, we used flow cytometry to quantify the proportion of poly(GP)-containing cells of dissociated *Tg(mnx1:gal4)/Tg(UAS:RFP)* 50 hpf embryos (Figure 2G, H). First, we confirmed the general accumulation of poly(GP) in the *c9orf72* KD condition when compared to the control, as shown by the significantly increased proportion of GFP-positive items in “all cells” and in “motor neurons” populations (Figure 2G, H). Furthermore, we found out that the proportion of poly(GP)-containing cells from *c9orf72* KD + poly(GP) embryos is significantly greater in the “motor neuron” than in “all cells” category, as showed in Figure 2G, H, thus demonstrating that motor neurons are more susceptible to poly(GP) accumulation under the genetic context of *c9orf72* loss of function.

SQSTM1/p62 is a standard component of neuronal and glial cytoplasmic inclusions that characterize many neurological disorders, including ALS and frontotemporal dementia (FTD) [45,46]. Particularly, an established hallmark of C9ALS-FTD pathology is the aggregation of DPRs in SQSTM1/p62-positive aggregates, as detected in postmortem tissues [5,6,40]. Therefore, we performed immunohistochemistry studies on transversal sections of *Tg(mnx1:gal4)/Tg(UAS:RFP)* 50 hpf embryos in order to detect Sqstm1/p62 and poly(GP) expression in spinal motor neurons. We observed that both Sqstm1/p62 and poly(GP) dipeptide specifically aggregate in motor neurons of *c9orf72* KD + poly(GP) condition when compared to the mismatch + poly(GP) control condition, as illustrated by images of sections in Figure 2I and quantified in Figure 2J. SQSTM1/p62 is an autophagy receptor that recognizes ubiquitinated cargo and targets it to the autophagy machinery, undergoing degradation in the process with the completion of the autolysosome fusion [47]. Subsequently, aggregated SQSTM1/p62 can signal incomplete processing of the autophagy cargo [48], potentially a central pathological mechanisms of ALS [49–51]. Of interest, mutations of *SQSTM1* have been reported in around 1–3.5% of familial ALS patients [52]. To further define the contribution of autophagy deficits to the accumulation of poly(GP), we explored the functional link between a decreased expression of the autophagy receptor Sqstm1/p62 and poly(GP)-related toxicity. For this, we used a previously established dose of a specific anti-*sqstm1* AMO [42] that by itself does not elicit morphological defects or motor phenotypes in zebrafish larvae, as shown in Fig. S3H, I. However, TEER testing in 50 hpf zebrafish show that combining partial knockdown of *sqstm1* with poly(GP) (*sqstm1* KD + poly(GP)) results in partial paralysis, as demonstrated by individual evoked swimming traces (Figure 2K), decreased distance (Figure 2L) and velocity (Figure 2M) parameters, when compared to non-injected and mismatch + poly(GP) control conditions. Overall, these results confirm that partially impairing autophagy by *sqstm1* knockdown is sufficient to phenocopy the effect of *c9orf72* KD on relative poly(GP) toxicity.

### Autophagy defects underlie the phenotoxic effect of C9orf72 gain and loss of function

**Figure 3.**
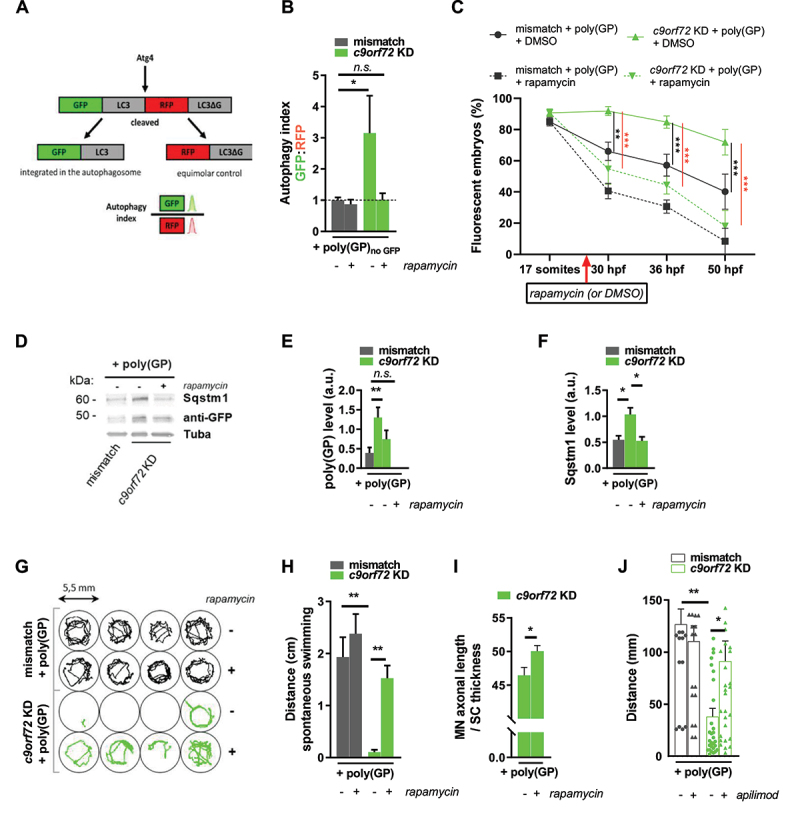
Autophagy induction rescues the synergistic toxicity of *C9orf72* gain and loss of function.

Note: (**A, B**) Monitoring of autophagy flux using GFP-LC3-RFP-LC3Δ fluorescent probe. (**A**) The autophagy probe is injected in zebrafish embryo at one-cell stage. It is cleaved by endogenous ATG4 proteases into equimolar amounts of GFP-LC3 and RFP-LC3ΔG. GFP-LC3 is integrated in the autophagosome and degraded by the lysosome, while RFP-LC3ΔG remains in the cytosol, and serves as an internal control. Autophagy flux is quantified by flow cytometry through GFP:RFP signal ratio of 50 hpf dissociated zebrafish. (**B**) Quantification of autophagy index showing reduced efficiency of autophagy activity in *c9orf72* KD + poly(GP)_no GFP_ zebrafish which is restored by rapamycin treatment. (**C**) Quantification of zebrafish displaying GFP fluorescence as a reporter of poly(GP) expression over different time points, showing that poly(GP) increased expression in *c9orf72* KD + poly(GP) condition is countered by rapamycin treatment. (**D-F**) Immunoblot (**D**) and quantifications of poly(GP) (**E**), and Sqstm1/p62 (**F**) proteins levels relative to Tuba/α-tubulin, showing that rapamycin treatment alleviates their accumulation. (**G, H**) Automated detection of spontaneous swimming of 72 hpf zebrafish following rapamycin (or DMSO) treatment (1 min time bin). Representative traces (**G**) and average distance (**H**) of spontaneous swimming showing that rapamycin treatment rescues the locomotor phenotype of *c9orf72* KD + poly(GP) zebrafish. (**I**) Rapamycin treatment rescues motor neuron axonal morphology of 50 hpf zebrafish as measured per average motor neuron (MN) length to dorsal (SC) thickness. (**J**) TEER results showing that apilimod treatment improves the average distance of *c9orf72* KD + poly(GP) 50 hpf zebrafish. * p < 0.05; ** p < 0.01; *** p < 0.001. Data are presented as mean + SEM (or mean ± SEM in **E**).

To test autophagy flux in our model, we used the GFP-LC3-RFP-LC3ΔG fluorescent probe [53] which is cleaved by endogenous Atg4 when autophagy is induced, thus giving rise to equimolar amounts of GFP-LC3 and RFP-tagged LC3ΔG mutant, which, unlike the full-length LC3, cannot be lipidated [54]. Upon autophagy activation, GFP-LC3 is integrated in the membrane of the autophagosome and is degraded by lysosomal proteases, whereas RFP-LC3ΔG remains in the cytosol and acts as an internal control (Figure 3A). Thus, the GFP/RFP fluorescence ratio (termed “autophagy index”) represents a live read-out of autophagy process, its value inversely correlated to the efficiency of autophagy [53]. To determine the autophagy index in our *c9orf72* model, we expressed the probe by injection at the 1-cell stage embryo and performed flow cytometry analysis of fluorescent cells from dissociated 50 hpf zebrafish, as previously described [53]. For this experiment we have used the poly(GP) construct with no GFP tag, poly(GP)_noGFP,_ to avoid the interference with the fluorescent probe. We observed that the autophagy index is significantly increased in *c9orf72* KD + poly(GP)_noGFP_ condition when compared to the control, thus indicating a failure of autophagy completion in this condition (Figure 3B), while no difference was observed between mismatch and *c9orf72* KD control conditions (in the absence of poly(GP)) (Fig. S4A). Importantly, rapamycin treatment is capable to restore the autophagy index of *c9orf72* KD + poly(GP)_noGFP_ zebrafish embryos down to normal values (Figure 3B). To note, we observed that rapamycin treatment did not decrease autophagy index in control conditions (Figure 3B and Fig. S4A), while bafilomycin A_1_ increases this ratio (Fig. S4A), indicating that autophagy is active in these basal conditions, as it has been previously shown in the development of zebrafish [55–58]. We then monitored the effect of rapamycin treatment on poly(GP) clearance at different stages of development: 17 somites, 30 hpf, 36 hpf and 50 hpf (Figure 3C). The poly(GP) expression is comparable between mismatch and *c9orf72* KD conditions at early stages of development, as shown at the 17 somites stage (Figure 3C). However, between 30 hpf to 50 hpf, mismatch + poly(GP) and *c9orf72* KD + poly(GP) embryos display significant differences in poly(GP) clearance (Figure 3C). In the *c9orf72* KD condition, rapamycin treatment starting at 24 hpf mitigated the accumulation of poly(GP), leading to a significant decrease in the expression of poly(GP) at 30 hpf, 36 hpf and 50 hpf stages when compared to the non-treated (Figure 3C). To confirm these observations, we performed immunoblotting on protein extracts from 50 hpf embryos, showing that autophagy activation with rapamycin improves poly(GP) clearance in the *c9orf72* KD condition (Figure 3D, E) and in the mismatch control conditions (Fig. S4B, C). Autophagy improvement in rapamycin treated *c9orf72* KD + poly(GP) zebrafish is confirmed by the reduction of Sqstm1/p62 accumulation to control levels (Figure 3D, F). Similar levels of SQSTM1/p62 are observed between mismatch + GFP and *c9orf72* KD + GFP control conditions and rapamycin treatment did not affect SQSTM1/p62 levels in these conditions (Fig. S4D, E). To test the correlation to the motor phenotype, spontaneous swimming after 2 days of rapamycin treatment was analyzed using an automated motion detection set-up. Consistent with the TEER results at 50 hpf, concomitant *c9orf72* KD and poly(GP) expression was associated with a reduction in spontaneous swimming parameters at 3 dpf, as shown by the traces of automated swimming recordings (Figure 3G) and by the quantification of the average distance covered by zebrafish embryos in Figure 3H. Activation of autophagy with rapamycin restored the motor abilities of *c9orf72* KD + poly(GP) zebrafish embryos, as compared to the non-treated condition (Figure 3G, H). This effect was associated with a rescue of the motor neuron axonopathic phenotype, as shown on the quantification of the axonal length in Figure 3I. Finally, to ascertain that the rescue obtained with rapamycin was related to a positive effect on autophagy, we treated zebrafish embryos with apilimod, a PIKFYVE inhibitor which was found to enhance the fusion of autophagosome and lysosome [59–61] and to improve survival of motor neurons derived from C9-ALS patients’ iPSC [62]. In our model, we observed that apilimod treatment also improves *C9orf72* pathology, as shown by the increased distance traveled travelled by treated *c9orf72* KD + poly(GP) embryos during TEER test (Figure 3J), thus confirming the role of autophagy dysfunction in C9orf72 pathogenesis.

### Synergy of C9orf72 gain and loss of function alters mitochondrial function in motor neurons

**Figure 4.**
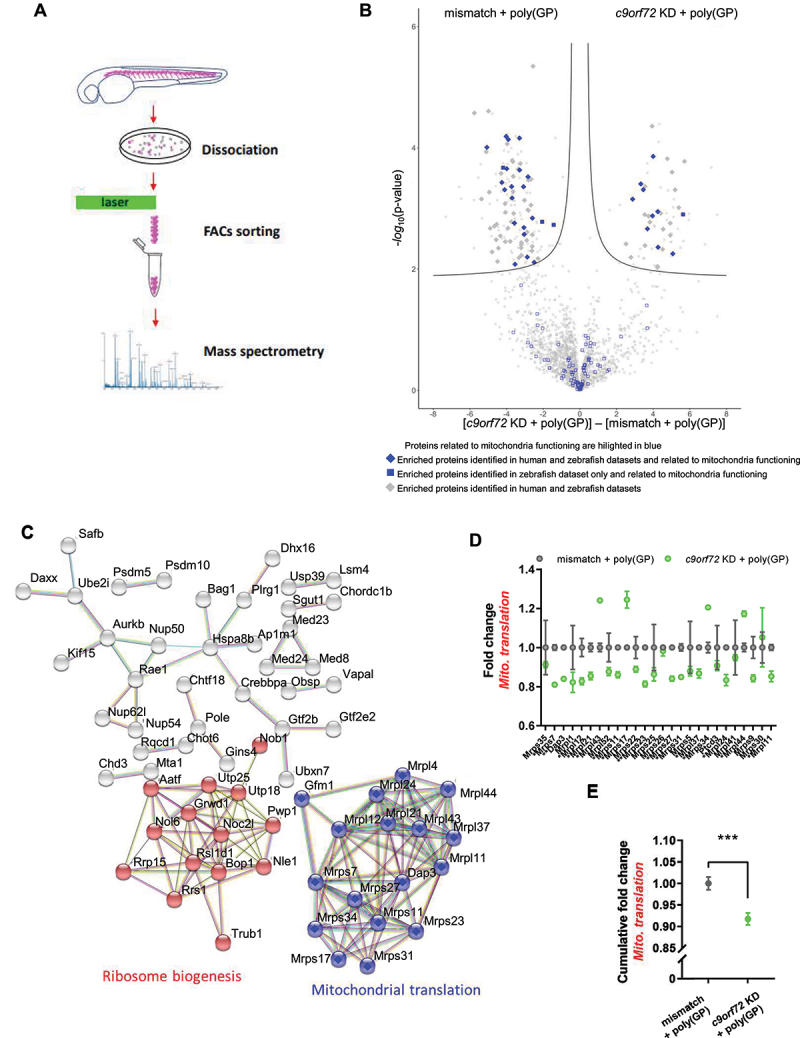
Proteomics indicates deficit in mitochondria function in motor neurons of *C9orf72* gain and loss of function zebrafish.

Note: (**A**) Purification of motor neurons by FACS sorting of dissociated transgenic *Tg(mnx1:gal4)/(UAS:RFP)* zebrafish for mass spectrometry analysis. (**B**) Differential proteomes of mismatch + poly(GP) and *c9orf72* KD + poly(GP) morphants. Volcano plot represents the statistical comparison of the proteome of *c9orf72* KD + poly(GP) versus mismatch + poly(GP) (control). Volcano plot was established using S0 = 0.1, FDR = 0.05. The abscissa reports the fold change in logarithmic scale (difference), the ordinate the -log(p-value). Proteins related to mitochondria functioning are highlighted in blue. All proteins found in common in human and zebrafish datasets are represented with a diamond shape. Proteins related to mitochondria and exclusive to zebrafish are highlighted with blue square symbol and proteins common in zebrafish and human datasets are represented with blue diamond symbol (filled: significant, unfilled: non-significant). Other homologous found in human are represented with graygrey diamond symbol (filled: significant, unfilled: non-significant). Finally, protein only found in zebrafish dataset was represented with small graygrey empty square symbol. (**C**) Network analysis of the 223 significantly dysregulated zebrafish proteins found through our proteomics analysis. STRING network analysis using *Danio rerio* database and highest confidence (0.900) as a minimum required interaction score with a 5% FDR stringency. Disconnected nodes in the network were hidden and structure previews inside network bubbles was disabled. Significantly differentially enriched proteins found in both human and zebrafish datasets are indicated with a full diamond symbol. The cluster in blue corresponds to *mitochondrial translation* (CL:27039), the cluster in red corresponds to *ribosome biogenesis* (CL:27377). (**D**) Fold change for each of the 26 identified proteins involved in mitochondria translation (as identified by STRING cluster). Stars indicate the significantly enriched proteins. (**E**) Cumulative change in the abundance of proteins involved in mitochondria translation. Student’s t-test q value *<0.05; **<0.01; *** p < 0.001. Data are presented as mean ±+/- SEM.

To understand the mechanisms underlying motor neuron loss in the context of *C9orf72* mutation, we used the *Tg(mnx1:gal4)/Tg(UAS:RFP)* genetic background to purify motor neurons by fluorescence-activated cell sorting/FACS and perform a proteomics analysis (Figure 4A). Among the 2500 proteins that we were able to identify in the zebrafish database, we identified 223 proteins that were differently enriched between *c9orf72* KD + poly(GP) and the control mismatch + poly(GP) conditions (Figure 4B, Table S1). Interestingly, our proteomics analysis unraveled highly decreased levels of stathmin 1 (stmn1) in the zebrafish motor neurons undergoing C9orf72 pathology (Table S1). Furthermore, we observed that proteins related to mitochondria signaling are strongly and predominantly represented in the differentially enriched proteins, as identified by gene ontology and gene set enrichment analysis databases (Figure 4B, Table S1), indicating the involvement of this pathway in purified motor neurons neurodegenerative process.

From the network analysis, two network clusters were identified (“STRING clusters”), one specific to mitochondrial translation and the other to ribosome biogenesis (Figure 4C, Table S2). Of the 26 proteins identified in zebrafish motor neurons proteome as involved in mitochondria translation, 15 proteins are significantly changed between mismatch + poly(GP) and *c9orf72* KD + poly(GP) conditions (Figure 4D). From the proteomics analysis, we tested antibodies for the zebrafish orthologs of some of the MRPL and MRPS proteins that were deregulated, including MRPL11, MRPL12, MRPL37, MRPL44, MRPS23, MRPS27, MRPS34 in spinal cord sections. Following validation by immunocytochemistry of these antibodies using the reporter line for motor neurons, *Tg(mnx1:gal4)*/*Tg(UAS:RFP)*, we performed immunofluorescence labeling of two upregulated (MRPL44 and MRPS34) and two downregulated (MRPL12 and MRPL37) mitochondrial translation proteins identified by proteomics analysis on spinal cord transversal cryosections. We show that the zebrafish orthologs of these mitochondrial translational markers, Mrpl12 (mitochondrial ribosomal protein L12), Mrpl37, Mrpl44 and Mrps34, identifed through our proteomics analysis, were significantly dysregulated in *c9orf72* KD + poly(GP) motor neurons as compared to the non-phenotypic mismatch + poly(GP) and GFP control conditions (Fig. S5A, B), thus validating the proteomics analysis for mitochondrial translation markers (Figure 4D). Furthermore, cumulative change of the proteins involved in mitochondrial translation indicates a global decrease in *c9orf72* KD + poly(GP) motor neurons as compared to the control (Figure 4E), highlighting the reduced abundance of mitochondrial translation proteins in *c9orf72* KD + poly(GP) MN. Supporting the role of C9orf72 in mitochondria homeostasis, co-immunostaining of C9orf72, poly(GP) and Mrpl12 in transversal sections of zebrafish spinal cord suggest that C9orf72 signal partly colocalizes with poly(GP) and that part of c9orf72 and poly(GP) signals localize to the mitochondria (Fig. S5C).

Interestingly, mitochondria translation and ribosome biogenesis were also previously identified to be the most enriched functions in a proteomics analysis of *C9orf72*-ALS iPSC [63]. To further elucidate the deregulated pathways in these models, we performed a comparative analysis of zebrafish motor neurons of *c9orf72* KD + poly(GP) and *C9orf72*-iPSC proteomes and defined enrichment of shared mitochondrial and ribosomal processes in the comparative dataset (Figure 4B, C and Table S3). Specifically, we mapped human orthologs for the 223 significantly altered zebrafish proteins with 184 unique proteins matched against both datasets (i.e. zebrafish and human iPSC) (Table S2). We found that 91 significantly modified proteins from the human study overlap to the zebrafish dataset and among them, 94.5% (86 proteins) were significantly modified (Figure 4B). Furthermore, the STRING analysis of these shared proteins also emphasizes the strong enrichment of mitochondrial function (Figure 4C) (Table S2).

### Synergistic toxicity of C9orf72 gain and loss of function alters mitophagy

**Figure 5.**
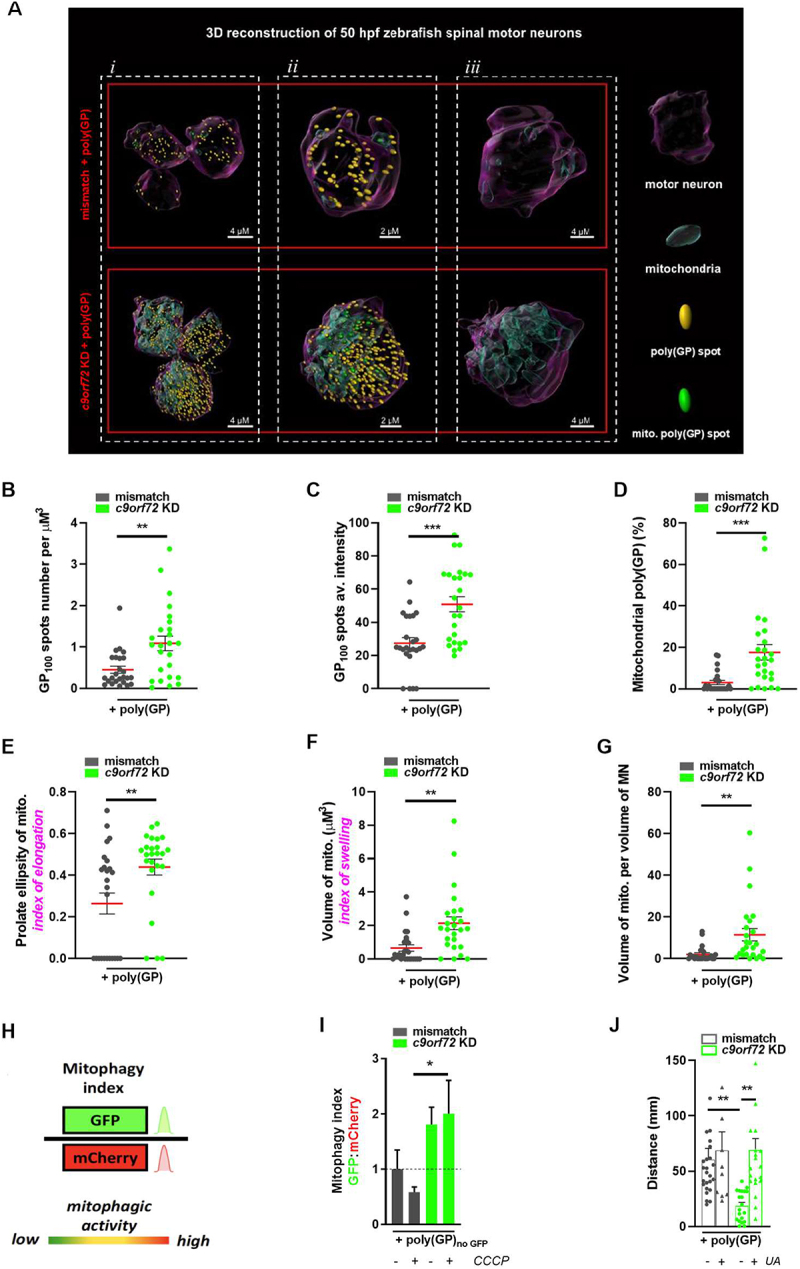
Mitophagy is altered under the synergistic effects of *C9orf72* gain and loss of function.

Note: (**A-G**) Results from 3D reconstructions of motor neurons from spinal sections of *Tg(mnx1:gal4)/(UAS:RFP)* 50 hpf zebrafish. (**A**) Mitochondria were identified with Tomm20 labeling and are pictured by cyan 3D structures. poly(GP) aggregates were identified with GFP labeling and are pictured by yellow spots, or by green spots when they colocalize to mitochondria. Representative images of 3D reconstructions are given for (***i***) three motor neurons, (***ii***) a zoom-in view of a motor neuron, and (***iii***) a zoom-in view of a motor neuron with mitochondria structures only. (**B-D**) Quantification of poly(GP) spots number per motor neuron volume (**B**), average (av.) fluorescence intensity from poly(GP) signal (**C**) and quantification of the proportion of poly(GP) aggregates colocalizing to mitochondria (**D**) in motor neurons, showing poly(GP) accumulation as well as increased colocalization of poly(GP) spots with mitochondria in motor neurons of *c9orf72* KD + poly(GP) condition. (**E-G**) Analysis of mitochondria morphology indexes in motor neurons, showing enlarged mitochondria structures in *c9orf72* KD + poly(GP) condition. (**E**) Average index of elongation of mitochondria (mito.) was quantified by their average prolate ellipsity. (**F**) Average index of swelling of mitochondria was quantified by assessing the average volume of mitochondria in motor neurons. (**G**) This panel shows the average total volume of mitochondria per volume of motor neuron (MN). (**H, I**) Measurement of mitophagy flux using the mitoQC fluorescent probe. (**H**) The mitoQC probe is injected in zebrafish embryo at one-cell stage. Mitophagy flux is then quantified by obtaining the ratio of GFP:mCherry signals using flow cytometry on dissociated cells from 50 hpf zebrafish. (**I**) Quantification of mitophagy index from flow cytometry results of GFP:mCherry signals of 50 hpf dissociated zebrafish showing decreased mitophagy activity in *c9orf72* KD + poly(GP)_noGFP_ zebrafish treated with CCCP. (**J**) TEER results showing that treatment with the mitophagy activator urolithin A improves the average distance of 50 hpf zebrafish. * *p *< 0.05; ** p < 0.01; *** *p *< 0.001. Data are presented as mean ±+/- or + SEM.

To determine the functional implications of proteomics observations and since mitochondrial function is tied to morphology [64,65], we proceeded to the analysis of mitochondria structure. We immunostained transversal sections of *Tg(mnx1:gal4)/Tg(UAS:RFP)* 50 hpf embryos with a TOMM20 antibody in order to observe mitochondria in spinal motor neurons. We show that Tomm20 fluorescence is increased and displays an abnormal pattern in *c9orf72* KD + poly(GP) condition as compared to the mismatch + poly(GP) condition while no difference is observed between mismatch + GFP and *c9orf72* KD + GFP conditions (Fig. S6A). Further, we performed 3D reconstructions from confocal images of mismatch + poly(GP) and *c9orf72* KD + poly(GP) motor neurons in order to determine the extent of poly(GP) accumulation inside mitochondria and to analyze their structure in detail (Figure 5A and Videos S1 and S2). First, we confirmed that there is a preferential increase of poly(GP) aggregates in the motor neurons of *c9orf72* KD + poly(GP) zebrafish larvae, as shown by the increased number and signal intensity of visible spots (Figure 5A-C and Videos S1, S2). Importantly, we confirmed that poly(GP) partly localize to the mitochondria, and that poly(GP) accumulation in *c9orf72* KD + poly(GP) condition is associated with its increased localization within these organelles (Figure 5A, D and Videos S1, S2). We also defined the mitochondrial morphology as quantified by the prolate ellipsity and show that mitochondria from the *c9orf72* KD + poly(GP) motor neurons are significantly more elongated than motor neurons from the mismatch + poly(GP) condition, as Figure 5E. In addition, mitochondria of motor neurons undergoing *C9orf72* pathology are more numerous (Fig. S6B) and swollen as compared to the mitochondria of the control condition (Figure 5F). Finally, we reported the ratio of the average volume of mitochondria to the average volume of motor neurons which is significantly increased in *c9orf72* KD + poly(GP) conditions, as illustrated by Figure 5A, Videos S1, S2 and quantified in Figure 5G. To maintain mitochondrial quality, dysfunctional mitochondria are normally selectively sequestered and eliminated through their autophagy processing [66]. In light of the effect of *C9orf72* mutation on autophagy functioning, and given that SQSTM1/p62 is involved in targeting mitochondria to autophagosomes [67,68], we explored whether mitophagy is defective in our model. To answer this question, we used another binary-based fluorescent sensor, the “mito-QC” probe in order to monitor mitophagic flux in zebrafish [69,70] (Figure 5H). Mito-QC consists of a tandem mCherry-GFP tag fused to the mitochondrial targeting sequence of FIS1, a resident protein of the outer mitochondrial membrane. During mitophagy, the acidic environment of the lysosome quenches GFP fluorescence without influencing the mCherry signal. The GFP:mCherry fluorescence ratio calculated from flow cytometry analysis is an indicator of mitophagy efficiency, with higher ratios indicating lower mitophagy turnover. The poly(GP) construct used for this experiment was poly(GP)_noGFP_ as to avoid interference with the fluorescent probe signal. For this assay, we used dissociated 50 hpf embryos which we treated with carbonyl cyanide 3-chlorophenylhydrazone (CCCP), a mitochondrial uncoupler that is routinely used to induce mitophagy [71]. Cells dissociated from the *c9orf72* KD + poly(GP)_noGFP_ condition show a significantly higher GFP:mCherry index (Figure 5I) when compared to the mismatch + poly(GP)_noGFP_ condition, thus confirming that the mitophagy process is impaired in the combined *c9orf72* gain – and loss-of-function condition. On the other hand, we observed no difference between mismatch and *c9orf72* KD control conditions (in the absence of poly(GP)), including upon CCCP treatment (Fig. S6C). Urolithin A (UA) is a small natural compound known to activate mitophagy [72–74] that has been shown to be protective in amyloid-β (Aβ) and tau models of Alzheimer disease [74]. We tested the ability of this potent mitophagy inducer to modify the locomotor phenotype of the zebrafish model of C9-ALS. As shown in Figure 5J, UA treatment significantly improved the swimming ability in this model, as shown by the increased distance traveled travelled by UA-treated *c9orf72* KD + poly(GP) embryos during TEER test when compared to the non-treated condition.

### Apoptosis mediates motor neuron degeneration in C9orf72 pathology

**Figure 6.**
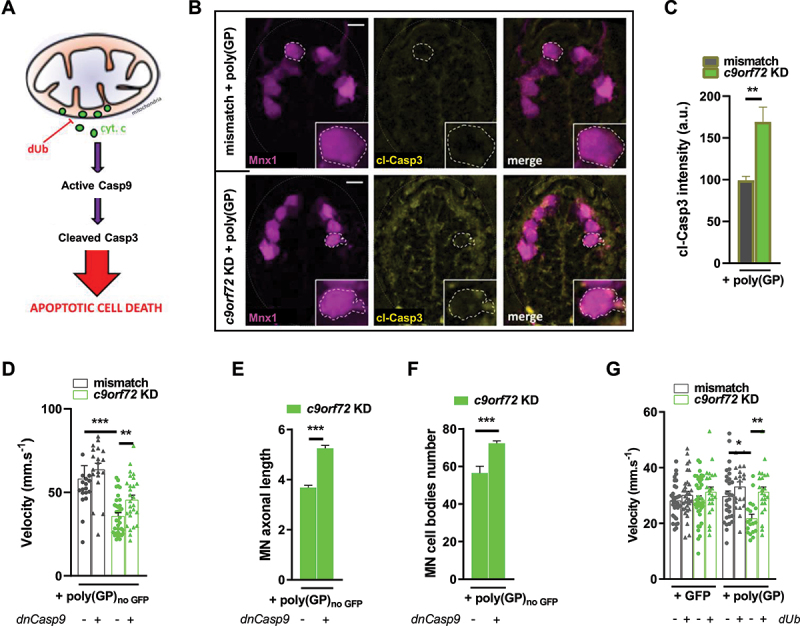
*C9orf72* pathology induces motor neurons apoptosis.

Note: (**A**) Schema of cytochrome c (cyt. c) release by mitochondria promotes Casp9 activation. Once activated, Casp9 goes on to cleave Casp3, initiating the caspase cascade of apoptosis. Decylubiquinone (dUb) is an inhibitor of the mitochondrial permeability transition pore. (**B, C**) Representative confocal images (**B**) and quantification of average fluorescence intensity (**C**) from cleaved Casp3 (cl-Casp3) immunolabeling in spinal sections of *Tg(mnx1:gal4)/(UAS:RFP)* 50 hpf zebrafish, showing increased fluorescence intensity in *c9orf72* KD + poly(GP) motor neurons. The dot lines encircle a representative motor neuron. A zoomed-in view is provided in the insert. Scale bar: 5 µM. (**D-F**) Analysis of the motor phenotype using triple transgenic *Tg(mnx1:gal4)/Tg(UAS:RFP)/Tg(UAS:GFP-dnCasp9)* 50 hpf zebrafish expressing a dominant negative dnCasp9 in motor neurons. Comparison of average velocity from TEER results (**D**), of average motor neuron (MN) axonal length to dorsal thickness ratio **(E**), and of average MN cell body number (**F**) of *c9orf72* KD + poly(GP)_noGFP_ 50 hpf zebrafish in triple transgenic dnCasp9-negative versus dnCasp9-positive conditions, showing that inhibiting capase-9 expression prevents the motor phenotype. (**G**) TEER results showing that dUb treatment improves the average velocity of 50 hpf zebrafish.

We observed that accumulation of dysfunctional mitochondria in motor neurons correlates with the degeneration of these cells leading to paralysis. Mitochondria being a key regulator of apoptosis activation, we hypothesized that mitochondria engage intrinsic apoptosis in motor neurons undergoing C9orf72 pathology. Typically, apoptosis starts with the cytosolic release of cytochrome c from the mitochondria (Figure 6A) [64,75,76]. This is followed by Casp9 (caspase 9) activation and subsequent cleavage of Casp3 (caspase 3), which initiates the caspase cascade that finally results in cell death (Figure 6A) [64,75,76]. To test our hypothesis, we performed immunohistochemistry on transversal sections of *Tg(mnx1:gal4)/Tg(UAS:RFP)* 50 hpf embryos to detect the activated (cleaved) form of Casp3. As shown in representative images of spinal cord in Figure 6B and quantified in Figure 6C, the intensity of the signal from cleaved Casp3 labelinglabelling is higher in *c9orf72* KD + poly(GP) condition than in mismatch + poly(GP) condition, while no difference is observed between mismatch + GFP and *c9orf72* KD + GFP conditions (Fig. S7). Further, we crossed the *Tg(mnx1:gal4)/Tg(UAS:RFP)* line with the *Tg(UAS:GFP-dnCasp9)* transgenic line, obtaining zebrafish embryos where a dominant negative GFP-tagged Casp9 was specifically expressed in motor neurons, thus inhibiting the caspase cascade in these cells [77]. We observed that motor neuron specific ablation of Casp9 function was protective of *C9orf72* gain and loss of function. Indeed, the TEER test revealed the improved velocity of dnCasp9-positive embryos, as compared with dnCasp9-negative embryos, from *c9orf72* KD + poly(GP)_noGFP_ condition (Figure 6D). Consistently, inhibiting Casp9 expression also prevents the degeneration of motor neurons, as shown by the significantly increased axonal length (Figure 6E) and survival of motor neurons (Figure 6F) in *c9orf72* KD + poly(GP)_noGFP_ embryos expressing the dominant negative form of Casp9. Finally, we tested the effect of decylubiquinone (dUb), an inhibitor of mitochondrial depolarization, which inhibits the release of Cycs (cytochrome c, somatic) and subsequent apoptosis (Figure 6A), on the locomotor performances of zebrafish embryos. Decylubiquinone is an analog of ubiquinone (coenzyme Q10), the latter having been reported to have beneficial effects in neurodegenerative disorders, including ALS [78]. In our model, dUb treatment ameliorated the motor behavior associated with *C9orf72* pathology, as shown by the increased velocity of treated *c9orf72* KD + poly(GP) embryos during TEER test (Figure 5G), thus confirming the causal relation between motor neuron degeneration and mitochondria-triggered apoptosis.

## Discussion

Along with other autophagy-related genes implicated in neurodegeneration and in ALS in particular, *C9orf72* has emerged in the last decade of research as an autophagy regulator [24,29,30,32,36,37,62,79–83]. C9orf72 was found to act at different steps of autophagy including the recruitment of ubiquitinated substrates, autophagosome formation and closure, as well as autophagosome-lysosome fusion and lysosome function [34]. Our results provide additional evidence confirming that dysregulated autophagy due to *C9orf72* mutation is an early event in ALS pathophysiology that enables the accumulation and subsequent toxicity of poly(GP). We demonstrate that low C9orf72 expression triggers poly(GP) and SQSTM1/p62 aggregation in motor neurons due to autophagy defects, as confirmed using the GFP-LC3-RFP-LC3ΔG fluorescent probe. In addition, combination of the partial inhibition of the autophagy receptor Sqstm1 and poly(GP) expression phenocopies the locomotor properties of c9orf72 gain and loss of function, thus supporting the specific involvement of autophagy in poly(GP) clearance. This is consistent with a recent study demonstrating that C9orf72 and SQSTM1/p62 have overlapping targets and associate in a common pathway of eliminating stress granules through the autophagic cascade [80]. It is becoming clear that autophagy deficiency is a relevant factor contributing to *C9orf72*/ALS pathogenesis, thus supporting the relevance of autophagy as a major therapeutic strategy to protect neurons from degeneration [84–86]. Here, we report that induction of autophagy at different levels has beneficial effects to counteract gain – and loss-of-function synergistic toxicity of *C9orf72* mutation. Indeed, activation of autophagy with rapamycin treatment compensates the loss of function of *c9orf72* as demonstrated by the specific clearance of poly(GP) and the restoration of motor neuronal structures and locomotor abilities. Furthermore, we also confirmed *in vivo* that targeting the later steps of autophagy with apilimod, a PIKFYVE inhibitor, to promote autophagosome and lysosome fusion, also ameliorates C9orf72 pathology, as previously observed in patient iPSC-derived motor neurons [62].

DPRs encoded from both the sense and the antisense strands of the C9orf72 have been shown to have different degrees of toxicity, with arginine-containing repeats, poly(PR) and poly(GR) appear to carry the highest toxic load when expressed at high levels in both animal and cellular models [87–89]. However, the relative abundance of these arginine-rich species of DPRs in patient tissue and biological fluids has been shown to be rather low [90–92], bringing into question their overall impact in the widespread motor neuron degeneration triggered by ALS. Paradoxically, one of the most abundant species of DPRs, poly(GP), the only one to be encoded by both forward and reverse hexanucleotide repeat strands, has been shown to carry no toxicity by itself in cellular and animal models of ALS [16–18]. However, accumulations of poly(GP), have been reported in the BAC-transgenic mice carrying C9orf72 repeats [93], and knockout of C9orf72 appears to accelerate the accumulation of poly(GP) [37], suggesting that autophagy dysfunction plays a role in its processing. In the zebrafish model presented we tested specifically the toxicity of poly(GP) in conditions that determine its accumulation. We show that a low level of expression of poly(GP) by itself is not intrinsically toxic in agreement with previous studies on GP. However, partial decrease of C9orf72 levels, or of autophagy processing, leads to widespread accumulation of poly(GP) associated with motor neuron degeneration. Interestingly, expression of similarly low levels of poly(GR) did not reach toxicity individually or in combination with an autophagy challenge, suggesting that at this level of expression, poly(GP) displays a particular sensitivity to C9orf72 levels, as well as autophagy defects, that can mediate its phenoconversion into a toxic species. These results could also suggest that arginine-rich DPRs, such as poly(GR), while carrying intrinsic toxic effects on a number of cellular pathways, including nucleocytoplasmic transport [94], mitochondrial function [95] and translation [96,97] are less sensitive to autophagy deregulation. This reinforces the concept that C9orf72 DPRs have distinct context and level dependent toxicity that can impinge on multiple pathways leading to neurodegeneration.

Accumulating evidence from the last decade of research on ALS points to a cumulative effect of the loss of function of C9orf72 on the toxicity of aggregation-prone proteins [29,37], including evidence for DPRs *in vitro* [36,62]. Furthermore, hexanucleotide repeat expansions were shown to enhance the phenotypic features, such as cognitive defects, activated astroglia and hippocampal neurodegeneration upon C9orf72 depletion in double-transgenic mice [37]. Similar to these findings, our results are consistent with the notion that poly(GP) undergoes a pro-pathological phenoconversion when C9orf72 levels are reduced. Still, it remains to decipher whether other preponderant DPR, such as poly(GA) also undergoes the same pathophysiological phenoconversion mechanism.

Our proteomics analysis revealed a downregulation of stmn1 in *C9orf72* KD + poly(GP) expressing motor neurons. STMN (stathmin) protein family is known to control microtubule dynamics and the regulation of their expression has been recently associated with motor neuron diseases, including ALS [98–101]. In particular, STMN2 (stathmin 2) is involved in motor neuron survival and axonal regeneration and known to be spliced by TARDBP/TDP-43, while STMN2 loss of function recently shown to directly contribute to TARDBP/TDP-43 pathology [98,99,101]. Therefore, deregulation of STMN1 could provide a potential missing link between C9orf72 gain and loss of function and TARDBP/TDP-43 related neurodegeneration.

Importantly, we observed that poly-GP accumulate and converge to mitochondria in motor neurons undergoing *c9orf72* KD. This feature is correlated with the presence of abnormally enlarged and swollen mitochondria, suggesting that mitochondrial dynamics and processing are disrupted. Indeed, healthy mitochondria normally undergo continuous remodeling by fusion and fission events [65]. Mitophagy processing is correlated with mitochondrial dynamics as it relies on fission to perform the engulfment and elimination of small and isolated mitochondria [102–105]. Interestingly, fission is mediated by DNM1L/Drp1, a protein recruited to mitochondria by FIS1 [106], which has been previously shown to genetically interact with C9orf72 [107]. Consistent with previous reports that identified C9orf72 protein in mitochondria-enriched fractions [108,109], we confirm that *C9orf72* pathology leads to mitophagy defects *in vivo*. To our knowledge, this is the first demonstration of an effect of *C9orf72* mutation on mitophagy process. Furthermore, proteomic analysis uncovered consistent abnormalities in mitochondrial components specifically in motor neurons, thus supporting the central role of mitophagy and mitochondrial deficits in this model of C9orf72 pathology. Importantly, these results matched previous analysis that was performed on iPSCs derived from patients carrying C9orf72 mutations, thus supporting mitochondrial homeostasishomoeostasis as a major pathway in pathogenesis [110,111]. Consequently, we found that treatment with urolithin A (UA) ameliorates the locomotor symptoms associated with *C9orf72* pathology. UA is an FDA-approved molecule that is known to improve mitochondrial proteostasis by inducing mitophagy [72–74,112].

Since little was known about the type of neuronal death engaged by the C9orf72 pathology, here we also confirmed the activation of the apoptotic pathway downstream of accumulating mitochondrial damage, identifying that motor neuron degeneration is associated with increased amount of cleaved Casp3, as previously reported in iPSC-derived motor neurons [113], and is dependent of Casp9 activity. Indeed, we demonstrate that neurodegeneration ensuing from apoptosis induction can be aborted by blocking Casp9 activity or by treating zebrafish with dUb. dUb is an FDA-approved drug, known to inhibit the mitochondrial permeability that precedes cytochrome c release in apoptosis pathway [114,115] and has been shown to improve motor features and survival in the mutant SOD1 transgenic mice [116]. dUb anti-apoptotic effect brings new therapeutic perspectives as it protects motor neurons in the final phase of the pathogenic cascade.

**Figure 7.**
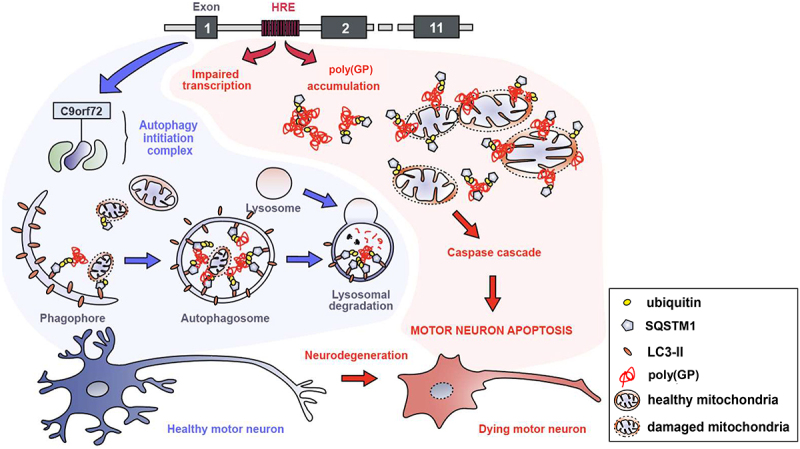
Proposed mechanisms for poly(GP) toxicity in C9orf72 haploinsufficiency. *C9orf72* knockdown induces poly(GP) and SQSTM1/p62 accumulation in motor neurons due to autophagy alteration. These synergistic effects of C9orf72 loss of function and poly(GP) expression perturb mitochondrial homeostasis, including mitophagy deregulation. Subsequently, accumulated abnormal mitochondria engage the cell death cascade through cascade activation, finally resulting in motor neuron degeneration through apoptosis cell death and arising motor deficits. Several drugs have been identified as being able to counteract motor deficits in this C9orf72 model: the MTOR inhibitor rapamycin and the PIKFYVE inhibitor apilimod, both by activating autophagy; the mitophagy activator urolithin A; and decylubiquinone, an analogue of ubiquinone (coenzyme Q10) and inhibitor of mitochondrial depolarization.

Overall, we describe here several interconnected cellular mechanisms that fail in *C9orf72* pathogenesis related to poly(GP) toxicity and converge toward motor neuron cell death mediated by apoptosis (Figure 7). We propose that dysregulated autophagy is an early event in the synergistic toxicity of *C9orf72* mutation that triggers accumulation of poly(GP) in motor neurons (Figure 7). A direct consequence of this event is a defective mitophagy, resulting in the accumulation of dysfunctional mitochondria and triggering the caspase cascade leading to motor neuron degeneration (Figure 7). Targeting of these pathways is a potential neuroprotective strategy for motor neurons in the context of *C9*-ALS and related neurological disorders.

### Study limitations

A major limitation of our study is the artificial expression of poly(GP) with a strong promoter. To mitigate for these effects, we performed a number of controls. In particular, we confirmed that removing the GFP reporter sequence, as well as removing the flag and the tag and moving the GFP reporter to the C-terminal end of GP repeats do not differ poly(GP) accumulation, MN degeneration and paralysis. However, we cannot exclude that the presence of these sequences can alter the molecular pathophysiology of poly(GP), as it has been shown recently for other overexpressed DPRs [117]. A more accurate methodology to model the *C9orf72* mutation would also be the introduction of HREs or poly(GP) repeats in the *C9orf72* locus. Generation of such stable transgenic models could allow us to determine whether aging could contribute to motor deficits as well as accumulation and/or aggregation of poly(GP). Despite these limitations, the consistency of proteomics results obtained from the motor neurons sorted from our zebrafish model with iPSCs from patients carrying *C9orf72* HRE, as well as the similar accumulation of poly(GP) assessed by immunoassay both in patient brain autopsy samples and zebrafish samples further support the proximity and translability of this zebrafish model, further our understanding of potential pathogenic mechanisms due to poly(GP) repeats and describes therapeutic strategies for ALS, FTD and related neurodegenerative diseases.

## Materials and methods

### Zebrafish maintenance

Adult and larval zebrafish (*Danio rerio*) were maintained at the Brain and the Imagine Institutes (Paris) fish facilities and bred according to the National and European Guidelines for Animal Welfare. Experiments were performed on wild-type and transgenic embryos from AB strains. *Tg(mnx1:gal4)icm11* [118] zebrafish were crossed with *Tg(UAS:RFP)* zebrafish to generate double transgenic zebrafish line having a specific expression of RFP in motor neurons allowing the observation of cell bodies of single motor neurons and their axonal arborization within a somatic segment in fixed and lived animals (hereinafter referred as *Tg(mnx1:gal4)/Tg(UAS:RFP)*.) *Tg(mnx1:gal4)/Tg(UAS:RFP)* zebrafish were crossed with *Tg(UAS:GFP-dnCasp9)* [77] to generate triple transgenic embryos expressing a GFP-fluorescent dominant negative (dn) Casp9 in RFP-fluorescent motor neurons in order to inhibit and label apoptosis initiation within those specific cells (hereinafter referred as *Tg(mnx1:gal4)/Tg(UAS:RFP)/Tg(UAS:GFP-dnCasp9)*). Zebrafish were raised in embryo medium: 0.6 g/L aquarium salt (Instant Ocean, SS15-10) in reverse osmosis water 0.01 mg/L methylene blue. Experimental procedures were approved by the National and Institutional Ethical Committees (26775). Embryos were staged in terms of *hours post fertilization* (hpf) based on morphological criteria [119] and manually dechorionated using fine forceps at 24 hpf. All the experiments were conducted on morphologically normal embryos.

### Microinjections

*AMOs –* Morpholino antisense oligonucleotides (AMOs; GeneTools, Philomath, USA) were used to specifically knockdown the expression of *c9orf72* and *sqstm1* genes. The AMOs were designed to bind to the ATG of zebrafish *c9orf72* and *sqstm1* orthologs. The sequences of *c9orf72* and *sqstm1* AMOs are respectively: 5’-ATTGTGGAGGACAGGCTGAAGACAAT-3’ and ATGAAGAGACGGAAAGTGTCATCCT-3’. *c9orf72* and *sqstm1* control AMOs, containing mismatch (mis) nucleotides with their respective ATG AMO sequences, and not binding anywhere in the zebrafish genome have the following sequences 5’-ATTcTcGAGcACAGcCTcAAGACAT-3’ and 5’ – ATcAAcAGACGGAAAcTcTCATCCT-3’. We used subphenotypic doses (half the effective dose) of 0.2 mM for *c9orf72* knockdown (and its control mismatch AMO) [25] and 0.3 mM for *sqstm1* knockdown (and its control mismatch AMO) [42]. *mRNA* – For rescue experiments, *C9orf72* mRNA encoding wild-type human long variant was microinjected at the final RNA concentration of 100 ng/μl as previously described [25]. *Plasmids* – All Poly(GP) and poly(GR) plasmids were kindly provided by Dr Nicolas Charlet-Berguerand [IGBMC, Illkirch, France]. For poly(GP) (10 and 50 repeats of glycine proline), poly(GP)_noGFP_ and poly(GR) (89 repeats of glycine arginine), constructs were cloned into the EcoRI and BamHI restriction sites of the pGFP vector (Clonetech, 632370). Poly(GP)_noTAG_ construct was cloned into the EcoRI and BamHI restriction sites of the pAAV-GFP vector. The sequences have been modified to evade pure GGGCC repeats. Respective GFP-only control plasmids were used as controls. Plasmids were microinjected at the DNA concentration of 100 ng/μl resulting in a final concentration of 0.03 µM. For the experiments involving the *Tg(mnx1:gal4)/Tg(UAS:RFP)/Tg(UAS:GFP-dnCasp9)* line, or the autophagy and the mitophagy probes, the poly(GP)_noGFP_ was microinjected at the final DNA concentration of 100 ng/μL. Optimal RNA and plasmid concentrations were determined as the point on the toxicity curve where there was no significant increase in the percentage of morphologically deformed larvae, an indication of overall toxicity. All the microinjections were carried out at one cell stage.

### Motor behavior of zebrafish embryos

Locomotor phenotypes of 50 hpf zebrafish embryos were assessed using the *Touched-Evoked Escape Response* (TEER) test, as previously described [25,41,42]. Briefly, embryos were touched on the tail with a tip and the escape response was recorded until the end of the swimming bout, using a Grasshopper 2 camera (Point Grey Research, Canada) at 30 frames per second. Distance and velocity parameters were quantified per each embryo for the entire duration of the swimming episode using the video tracking plugin of FIJI 1.47 software [120]. Spontaneous movements of 72 hpf zebrafish embryos were analyzed using an automated imaging and analysis system (Zebralab, ViewPoint, France). Single embryos were placed in individual wells of a 96-well plate and recorded. Distance and velocity parameters were computed using the Live Tracking module in 20-min intervals. In drug rescue experiments, 28 hpf zebrafish embryos were raised in embryo medium containing 0.5 µM rapamycin (Sigma, R0395) [41], 1 µM apilimod (Sigma, SML2974), 3 µM decylubiquinone (Sigma, D7911), or 5 µM urolithin A (Sigma, SML1791).

### Motor neuron morphology and survival

*Tg(mnx1:gal4)/Tg(UAS:RFP)* or *Tg(mnx1:gal4)/Tg(UAS:RFP)/Tg(UAS:GFP-dnCasp9)* embryos were used to image and quantify motor neurons features. For time-lapse fluorescence imaging of the primary motor neuron axonal growth, 19 hpf embryos were mounted in 1% low-melting-point agarose dissolved in embryo medium and supplemented with 0.16 mg/mL tricaine (Sigma, A-5040). Images were captured with a Spinning Disk system (Andor technology, UK; Leica Microsystems, Germany), a DMI8 inverted stand (Leica Microsystems, Germany), a CSU-X head (Yokogawa, Japan) and a QE-180 camera (Hamamatsu, Japan), with a 20X objective (NA 0.5) from 19 hpf to 33 hpf. Axonal lengths of four motor neurons ventral projections were measured using FIJI 1.47 [120] on images of growth periods of sixty min. Measures were taken from the ventral root to the ventral edge of the musculature within the fifteenth to the eighteenth segments. For primary and secondary axonal projections measurements and cell body counting, 50 hpf embryos were fixed in 4% paraformaldehyde and captured at the same defined location within the intersomitic segments with an Apotome.2 system and an Imager.M2 stand (Carl Zeiss, Germany), with a 20X objective (NA0.8). Motor neurons axonal length as well as spinal cord thickness were also measured with FIJI 1.47 [120]. Fluorescent cell bodies of primary and secondary motor neurons were counted within the same intersomitic segment region.

### Immunostaining on sections

*Tg(mnx1:gal4)/Tg(UAS:RFP)* or *Tg(mnx1:gal4)/Tg(UAS:RFP)/Tg(UAS:GFP-dnCasp9)* 50 hpf embryos were anaesthetized in 0.2% tricaine, fixed in 4% PFA and prepared for cryosections as previously described [121]. Samples were cut into 20-μm thick transversal sections which were blocked and permeabilized with 0.2% gelatin (Sigma, 04055), 0.25% Triton X-100 (Thermo Fisher Scientific, 85112) diluted in 1X PBS (Thermo Fisher Scientific, 11594516) and incubated overnight with primary antibodies: anti-GFP (1:500, chicken polyclonal; GeneTex, GTX13970), anti-SQSTM1 (1:250, mouse monoclonal; Santa Cruz Biotechnology, sc-28359), anti-TOMM20 (1:200, rabbit polyclonal; Sigma Aldrich, HPA011562), anti-cleaved CASP3 (1:250, rabbit polyclonal; Cell Signaling Technology, 9661), C9orf72 (1:50, mouse, provided by Dr Nicolas Charlet-Berguerand [IGBMC, Illkirch, France]), Mrpl12 (1:200, rabbit polyclonal; Proteintech, 14795), Mrpl44 (1:200, rabbit polyclonal; Proteintech, 16394), Mrps34 (1:100, mouse polyclonal; Sigma Aldrich, KPAO42112) and/or Mrpl37 (1:100, rabbit polyclonal; Sigma Aldrich, HPA025826). After several washes, sections were incubated 1 h with the appropriate secondary antibodies conjugated to an Alexa Fluor® (1:500; Thermo Fisher Scientific, A11039, A31556, A131553). Sections were rinsed and mounted in Fluoromount-G™ medium (Thermo Fisher Scientific, 00–4958-02). Images of spinal cord area were captured with a Spinning Disk system (Andor technology, UK; Leica Microsystems, Germany), a DMI8 inverted stand (Leica Microsystems, Germany), a CSU-X head (Yokogawa, Japan) and a QE-180 camera (Hamamatsu, Japan), with a 63X objective. Images were processed with FIJI 1.47 software [120], same treatments were applied for all the conditions, and average fluorescence intensities of two-dimensional images were measured in five motor neurons (or five regions of interest for C9orf72 immunostains) of at least three different embryos per condition. Reconstruction and analysis of three-dimensional images are detailed in *Three-dimensional reconstruction of motor neurons* paragraph below.

### Three-dimensional reconstruction of motor neuron structures

Images of *Tg(mnx1:gal4)/Tg(UAS:RFP)* 50 hpf embryos sections were pretreated on FIJI 1.47 [120] and segmented by machine learning using Ilastik software [122]. Images were then processed on Imaris 9.7.1 software (Oxford Instruments, UK). RFP-labeled labelled motor neurons were segmented manually with the surface module and colored in magenta. TOMM20-immunostained mitochondria structures were segmented with the surface module using the shortest distance calculation and colored in cyan. GFP-positive poly(GP) signal was segmented with the spots module using the shortest distance calculation and colored in yellow. A filter was applied to highlight and colored in green the poly(GP) spots colocalizing to the mitochondria structures. poly(GP) average intensity, percent of poly(GP) spots colocalizing to mitochondria, average prolate ellipsity of mitochondria, average volume of mitochondria, and average volume of mitochondria per motor neuron volume were quantified in five motor neurons of sections from five different embryos per each condition. Original signals are visible in **Videos S1 and S2**.

### Live imaging of poly(GP) expression

Embryos were screened for GFP fluorescence and counted at 17 somites, 30 hpf, 36 hpf and 50 hpf stages with a fluorescent binocular microscope (Olympus, Japan). For live imaging of whole mount embryos, 17 somites, 36 hpf and 50 hpf embryos were mounted in 1% low-melting-point agarose dissolved in embryo medium and supplemented with 0.16 mg/mL tricaine (A-5040, Sigma). Images were captured with a Spinning Disk system (Intelligent Imaging Innovations, USA), an Examiner.Z1 upright stand (Carl Zeiss, Germany), a CSU-W1 head (Yokogawa, Japan), and an ORCA-Flash 4.0 camera (Hamamatsu, Japan), with a 20x objective (NA1).

### Dissociation of zebrafish embryos

Dechorionated embryos were dissociated in EDTA-trypsin 0.25% at 28°C and by trituration. Digestion was stopped with 10% fetal calf serum and suspended cells were strained with a 40 µM strainer. Cells were then centrifuged (5 min at 0.8 g, 4°C) and washed with cold HBSS (Gibco^TM^, 14025092), twice. Cells were then resuspended in Leibovitz medium completed with 10% FBS, 1% L-glutamine and 2% Pen-Strep. Suspended cells were centrifuged at 0.8 g x g, 5 min and resuspended with the appropriate volume of the same medium. Cellular viability was assessed by flow cytometry using DAPI.

### Flow cytometry monitoring of autophagy and mitophagy flux

To monitor autophagy flux in zebrafish, we co-injected the GFP-LC3-RFP-LC3ΔG probe developed by Mizushima’s laboratory [53] at a final concentration of 120 ng/µL. To calculate the GFP:RFP ratio, we quantified the proportions of GFP-positive cells and of RFP-positive cells by flow cytometry of 50 hpf dissociated embryos, as previously established [53]. Rapamycin-treated dissociated cells were incubated in 1 µM rapamycin for 1 h. Bafilomycin A_1_ (Sigma, B1793)-treated cells were incubated with 50 nM bafilomycin A_1_ for 30 min. Similarly, to monitor mitophagy flux, we coinjected the Mito-QC probe [69] at a final concentration of 100 ng/µL and we calculated the GFP:mCherry ratio. Carbonyl cyanide 3-chlorophenylhydrazone (CCCP) -treated dissociated cells were incubated in 10 nM CCCP for 1 h. For both autophagy and mitophagy probes, cells were counted using a MACSQuant® Analyzer 10 Flow cytometer (Miltenyi Biotec, Germany). Dissociated cells from 50 hpf non injected embryos were used as a negative control for fluorescence and compensation was made with cells from dissociated 50 hpf embryos expressing GFP or RFP fluorescence only. Data were processed using FlowJo^TM^ software (BD, USA).

### Poly(GP) subcellular distribution and cell sorting of motor neurons

To analyze the distribution of poly(GP) in zebrafish embryos cells, *Tg(mnx1:gal4)/Tg(UAS:RFP)* 36 hpf embryos were dissociated as described above but resuspended in serum-free medium. GFP-positive and RFP-positive cells were counted, and RFP-positive cells were sorted, using a Sony SH 800S Cell Sorter cytometer (Sony, Japan). Compensation was calculated as described above and data were processed using FlowJo^TM^ software (BD, USA). Purified motor neurons were lysed in 5% SDS, 100 mM Tris-HCl buffer (pH 7.5) and proteins were kept at −80°C until spectrometry analysis.

### Proteomics analysis of motor neurons

S-TrapTM micro spin column (Protifi, C02-micro) digestion was performed on lysates of zebrafish motoneurons isolated by FACS. Proteins were alkylated with the addition of iodoacetamide to a final concentration of 50 mM. Aqueous phosphoric acid was added to a final concentration of 1.2%. Colloidal protein particulate was formed with the addition of 6 times the sample volume of S-Trap binding buffer (90% aqueous methanol, 100 mM TEAB (Thermo Fisher Scientific, 90114, pH 7.1). The mixtures were put on the S-Trap micro 1.7 mL columns and centrifuged at 4,000 g for 30s. The columns were washed four times with 150 µL S-Trap binding buffer and centrifuged at 4,000 g for 30s with 180 degrees rotation of the columns between washes. Samples were digested with 0.8 µg of trypsin (Thermo Fisher Scientific, 25200056) at 47°C 1 h 30 min. Samples were resuspended in 21 µL of 10% ACN, 0.1% TFA in HPLC-grade water. Each sample was injected three times. For each run, 5 µL was injected in a nanoRSLC-Q Exactive PLUS (RSLC Ultimate 3000) (Thermo Scientific, USA). Peptides were loaded onto a µ-precolumn (Acclaim PepMap 100 C18, cartridge, 300 µm i.d.×5 mm, 5 µm; Thermo Scientific, 160454), and were separated on a 50 cm reversed-phase liquid chromatographic column (0.075 mm ID, Acclaim PepMap 100, C18, 2 µm; Thermo Scientific, 164942). Chromatography solvents were (A) 0.1% formic acid in water, and (B) 80% acetonitrile, 0.08% formic acid. Peptides were eluted from the column with the following gradient 5% to 40% B (120 min), 40% to 80% (1 min). At 121 min, the gradient stayed at 80% for 5 min and, at 126 min, it returned to 5% to re-equilibrate the column for 20 min before the next injection. One blank was run between each replicates to prevent sample carryover. Peptides eluting from the column were analyzed by data dependent MS/MS, using top-10 acquisition method. Peptides were fragmented using higher-energy collisional dissociation (HCD). Briefly, the instrument settings were as follows: resolution was set to 70,000 for MS scans and 17,500 for the data dependent MS/MS scans in order to increase speed. The MS AGC target was set to 3.106 counts with maximum injection time set to 200 ms, while MS/MS AGC target was set to 1.105 with maximum injection time set to 120 ms. The MS scan range was from 400 to 2000 m/z. Dynamic exclusion was set to 30 seconds duration. The MS files were processed with the MaxQuant software version 1.6.14.0 and searched with Andromeda search engine against the UniprotKB/Swiss-Prot Danio rerio database (release 15/04/2019, 3126 entries). To search parent mass and fragment ions, we set a mass deviation of 3 ppm and 20 ppm respectively. The minimum peptide length was set to 7 amino acids and strict specificity for trypsin cleavage was required, allowing up to two missed cleavage sites. Carbamidomethylation (Cys) was set as fixed modification, whereas oxidation (Met) and N-term acetylation were set as variable modifications. The false discovery rates (FDRs) at the protein and peptide level were set to 1%. Scores were calculated in MaxQuant as described previously [123]. The reverse and common contaminants hits were removed from MaxQuant output. Proteins were quantified according to the MaxQuant label-free algorithm using LFQ intensities; protein quantification was obtained using at least 2 peptides per protein. Match between run was allowed. Statistical and bioinformatic analysis, including heatmaps, profile plots and clustering, were performed with Perseus software (version 1.6.7.0) [124]. For statistical comparison, we set two groups, *c9orf72* KD + poly(GP) (“C9”) and mismatch + poly(GP) (“Mis”), each containing biological triplicate. Each sample was run in technical triplicates as well. We then filtered the data to keep only proteins with at least 3 valid values in at least one group. Next, the data were imputed to fill missing data points by creating a Gaussian distribution of random numbers with a standard deviation of 33% relative to the standard deviation of the measured values and 2.5 standard deviation downshift of the mean to simulate the distribution of very low signal values. We performed a T-test, FDR = 0.05, S0 = 0.1. Protein interaction analysis of the enriched expressed proteins was done using String database with a minimum required interaction score of 0.900 (highest confidence). We reported experimentally determined interactions only. 223 significantly enriched zebrafish proteins were searched on Ensembl for their orthologous human genes counterpart using their respective gene name. A human orthologous gene was found for each of the 223 significant zebrafish proteins. Then, orthologous genes were mapped against human proteomics results matrix from previously published C9-ALS iPSC proteome [63] to analyze the overall overlapping between both studies.

### Western blot

50 hpf zebrafish embryos (15–30 per tube) were homogenized in lysis buffer: 50 mM Tris-HCl, pH 7.4, 500 mM NaCl, 5 mM EDTA, 1% (w:v) Nonidet P-40 (Roche, 11754599001), 0.5% (w:v) Triton X-100 supplemented with a cocktail of protease inhibitors (Complete mini; Roche, 11836153001). Whole tissue lysates were then centrifuged at 14000 × g at 4°C for 20 min. Supernatants were collected and frozen at −80°C until biochemical analysis. Thirty μg of proteins from 50 hpf embryos homogenized lysates were separated by SDS polyacrylamide gel electrophoresis. Samples were denatured at 98°C for 7 min. Separated proteins were transferred to nitrocellulose membranes (Bio-Rad, 1704270) and probed with the following primary antibodies: anti-GFP CHIP Grade (Abcam, ab290), anti-C9RANT (Novus BioTechne, NBP2-25018) and anti-SQSTM1 (Santa Cruz Biotechnology, sc-28359). Anti-GAPDH (Abcam, ab8245) and anti-TUBA/α-tubulin (Sigma, T5168) antibodies were used as loading controls. The blots were incubated with the corresponding fluorescent secondary antibodies (Li-Cor, 926–32,213, 926–32,212, 926–68072) and the signal was detected using an ODYSSEY® CLx system (Li-Cor, USA). The intensity of bands was measured with FIJI 1.47 [120].

### Poly(GP) immunoassay

RIPA soluble and insoluble fractions of four experimental replicates of twenty 50 hpf zebrafish embryos or postmortem human brains from occipital cortex and cerebellum (collected and provided by the Neurobiobank of Munich according to the guidelines of the ethical committee at the Medical Faculty of Ludwig-Maximilians-University Munich) were prepared as described previously, using a homogenizer (Precellys, 432–0478) [125]. Samples were first homogenized in RIPA buffer (137 mM NaCl, 20 mM Tris pH 7.5, 10% glycine, 1% Triton X-100, 0.5% Na-deoxycholate, 0.1% SDS, 2 mM EDTA); supplemented with protease (Sigma, P8340) and phosphatase (Sigma, P0044) inhibitors and benzonase nuclease (Sigma, E1014), then incubated while shaking at 4°C for 20 min and centrifuged at 13,000 g for 10 min at 4°C. To avoid cross-contamination between soluble and insoluble fractions, pellets were resuspended in RIPA, re-homogenized, and re-centrifuged. The RIPA-insoluble pellets were homogenized in RIPA buffer containing 3.5 M urea (U-RIPA), and protein concentration was determined by BCA assay. Poly(GP) immunoassay was performed to measure soluble and insoluble poly(GP) on the MSD platform using streptavidin plates (MSD Gold 96-well Streptavidin; SECTOR, L15SA) as described previously using rat monoclonal anti-GP antibodies 18H8 and 3F9 [43].

### Statistical analysis

Data were plotted and analyzed using Prism (GraphPad, USA). Normality was determined by Shapiro-Wilke test and homoscedasticity was tested with Levene’s test before analysis of variance. Locomotor data were analyzed by one or two-way ANOVA followed by Tukey’s multiple comparison. Axonal growth was analyzed by two-way ANOVA with repeated measures followed by Sidak’s multiple comparison. Morphology and survival of motor neurons were analyzed by one-way ANOVA followed by Tukey’s multiple comparison or by unpaired two-tailed t-tests. Protein levels from western blot experiments were analyzed by unpaired bilateral t-tests or by one-way ANOVA followed by Tukey’s multiple comparison. Cytometry data were analyzed by one or two-way ANOVA followed by Tukey’s multiple comparison. Fluorescence intensity from immunolabeling experiments and mitochondria morphology parameters were analyzed by unpaired bilateral t-tests or by one-way ANOVA.

## Supplementary Material

Supplemental Material

## Data Availability

Proteomics data set is available https://www.ebi.ac.uk/pride/archive/projects/PXD023967.
