# Supplementary material for: Poly-GP accumulation due to C9orf72 loss of function induces motor neuron apoptosis through autophagy and mitophagy defects
Source: Autophagy. 2024 Sep 24;20(10):2164–85. doi: 10.1080/15548627.2024.2358736 (PMC11423671; doi:10.1080/15548627.2024.2358736)
Supplement: Supplemental Material [file KAUP_A_2358736_SM0989.zip › suppl/Supplementary Figures R6.docx]

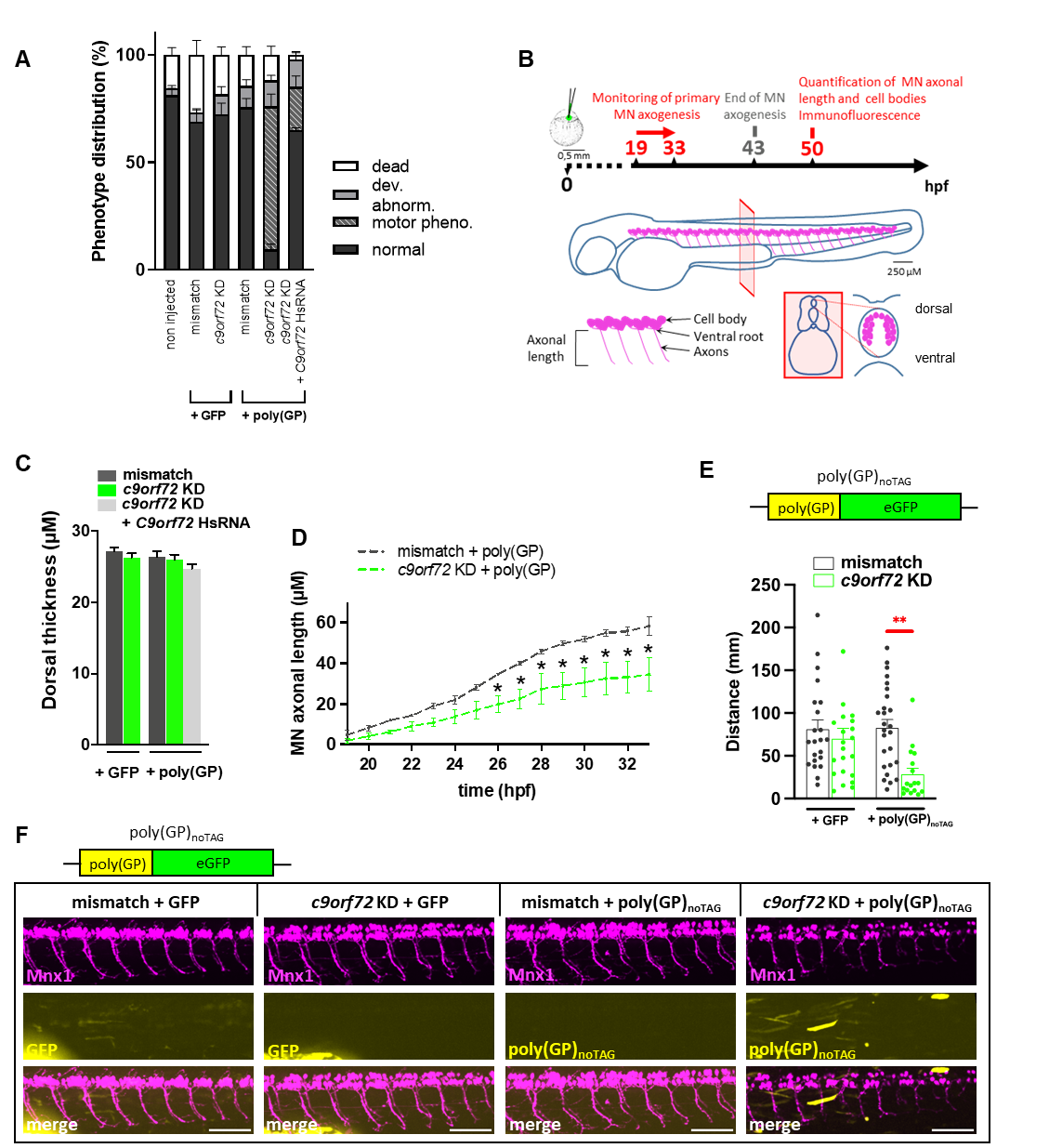


**Figure S1.** Poly(GP) induce motor neuron (MN) defects upon *c9orf72* knockdown (KD) (**A**) Distribution of phenotypes showing the proportion of 50 hpf zebrafish displaying motor defects in the different conditions. Note that restoring C9orf72 expression with *C9orf72* hRNA increases the percentage of normal zebrafish**.** (**B**) Double-transgenic *Tg(mnx1:gal4)/(UAS:RFP)* zebrafish larvae expressing RFP fluorescence in motor neurons were used for *in vivo* monitoring of axogenesis, for the quantification of spinal MN axonal length and number, and for immunofluorescent labeling on transversal spinal cord sections. (**C**) Dorsal thickness is constant among conditions. (**D**) Monitoring of primary motor neuron axogenesis from 19 to 33 hpf showing deleterious effect of *c9orf72* KD + poly(GP) on the emerging MN axonal projections. (**E**, **F**) “Poly(GP)_noTAG_” construct (for “poly(GP)” construct see **Fig S2A**). (**E**) Quantification of average swimming distance reflecting the motor phenotype of *c9orf72* KD + poly(GP)_noTAG_ 50-hpf zebrafish. Each dot represents one embryo. (**F**) Representatives images of *Tg(mnx1:gal4)/(UAS:RFP)* 50-hpf zebrafish illustrating the disruption of MN axonal beam and a lower density of MN cell bodies in *c9orf72* KD + poly(GP)_noTAG_ condition, as well as the accumulation of fluorescence from poly(GP)_noTAG_ expression in this condition. Scale bar: 100 µm. * p<0.05 ; ** p<0.01. Data are presented as mean + sem or +/- sem in (**D**).

**Figure S2.** Poly(GP) properties are not affected by the GFP tag. (**A**) Poly(GP) and poly(GR) amino acid sequences. (**B, C**) Poly(GP)_noGFP_ construct. Quantification of average swimming distance (**B**) and velocity (**C**) from TEER assay of 50 hpf zebrafish showing that poly(GP) without GFP tag (poly(GP)_noGFP_) induces a decreased of distance and velocity parameters in *c9orf72* KD condition. *** p<0.001. Data are presented as mean + sem.


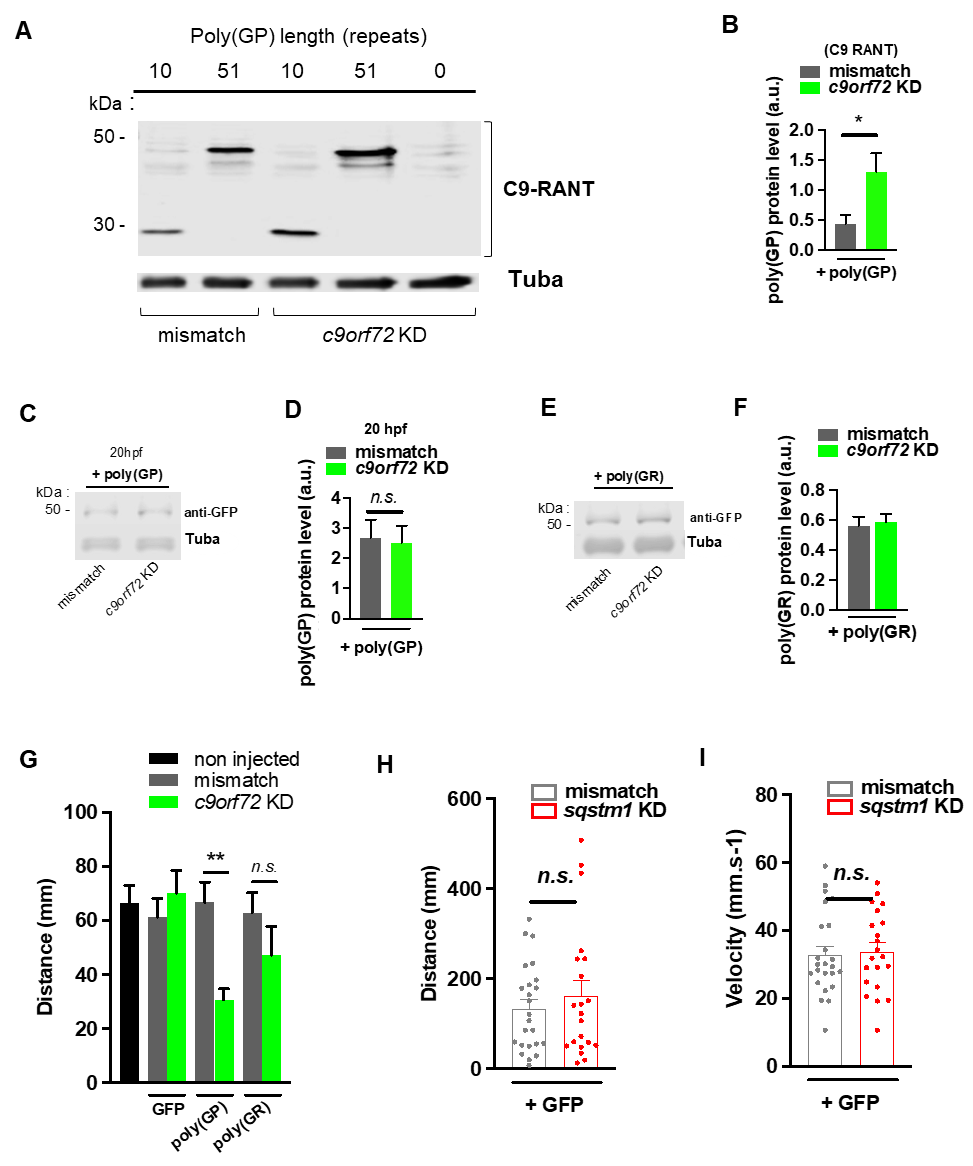


**Figure S3.** Poly(GP) effects are related to its accumulation. (**A**) Immunoblot of 50 hpf zebrafish lysates using C9 RANT antibody, showing poly(GP) accumulation in *c9orf72* KD conditions, including with a shorter length of poly(GP) (10 repeats). (**B**) Quantification of average poly(GP) levels relative to Tuba/α-tubulin at 50 hpf using the C9-RANT antibody showing increased levels of poly(GP) in *c9orf72* KD condition. (**C**, **D**) Immunoblot (**C**) and quantification of average poly(GP) levels relative to Tuba/α-tubulin (**D**) at 20 hpf using the anti-GFP antibody showing no difference between conditions. (**E**, **F**) Immunoblot (**E**) and quantification of average poly(GR) levels relative to Tuba/α-tubulin (**F**) at 50 hpf using the anti-GFP antibody showing no difference between conditions. (**G**) Quantification of average swimming distance from TEER assay showing that 50 hpf zebrafish expressing poly(GR) swim normally, including under *c9orf72* KD. (**H**, **I**) Quantification of average swimming distance (**H**) and velocity (**I**) from TEER assay showing no difference between mismatch + GFP and *sqstm1* KD + GFP conditions. *<p0.05; ** p<0.01; *n.s.* non significant. Data are presented as mean + sem.


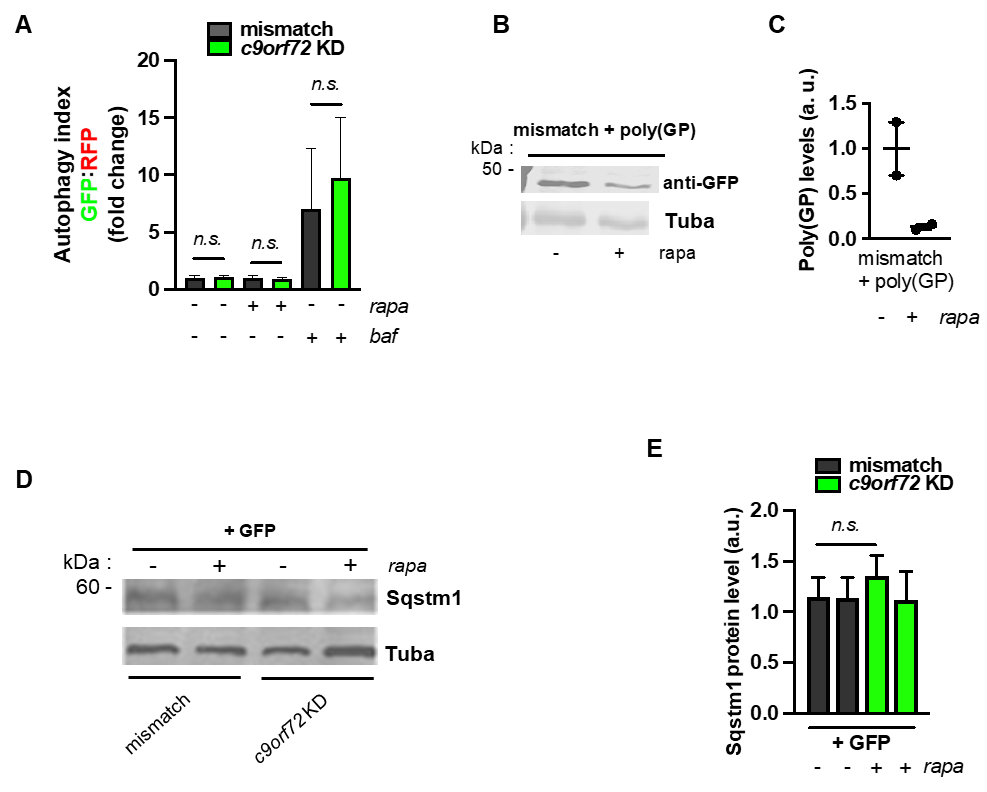
**Figure S4.** Autophagy activity is normal upon *c9orf72* KD in the absence of poly(GP). (**A**) Quantification of autophagy index of 50 hpf zebrafish showing no difference in autophagy activity between mismatch and *c9orf72* KD conditions (without poly(GP)). Dissociated cells were treated, or not, with rapamycin (rapa) or bafilomycin (baf), showing basal autophagy activity. (**B**, **C**) Immunoblot (**B**) and quantification (**C**) of average poly(GP) levels relative to Tuba/α-tubulin at 50 hpf using the anti-GFP antibody showing that rapamycin decreases poly(GP) levels in control condition. (**D**, **E**) Immunoblot (**D**) and quantification (**E**) of average Sqstm1/p62 protein levels relative to Tuba/α-tubulin at 50 hpf using the showing no difference between mismatch + GFP and *c9orf72 +* GFP control conditions. *n.s.* non significant. Data are presented as mean + sem.


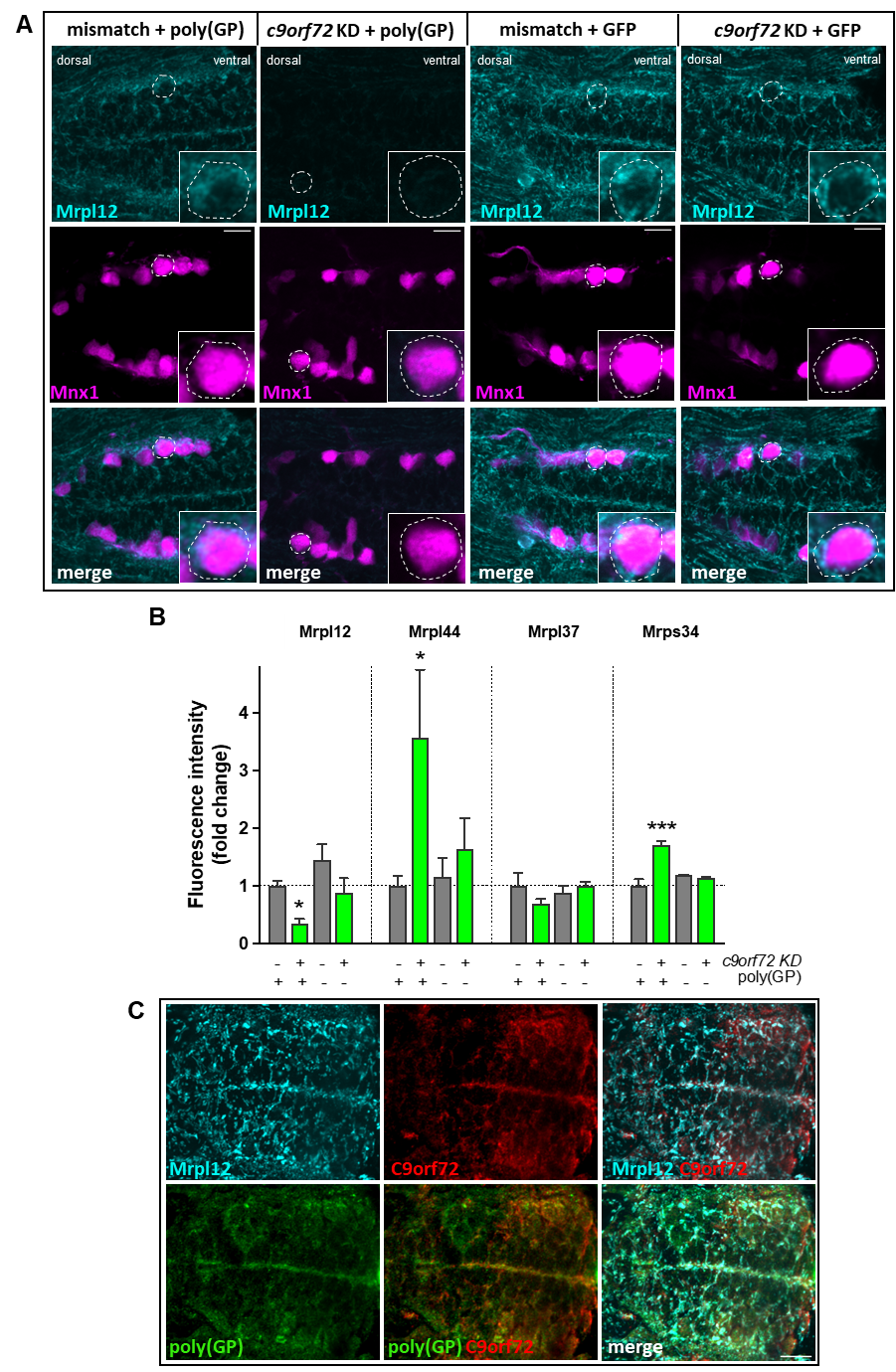


**Figure S5.** *c9orf72* KD + poly(GP) zebrafish motor neurons (MN) display abnormal mitochondria functioning. (**A**, **B**) Validation of mitochondrial translation proteins hits identified by proteomics. (**A**) Representative confocal images of Mrpl12 immunostains in spinal MN from transversal sections of *Tg(mnx1:gal4)/(UAS:RFP)* 50 hpf zebrafish. The dot lines encircle a representative motor neuron. A zoomed-in view is provided in the insert. (**B**) Quantification of average fluorescence intensity from Mrpl12, Mrpl44, Mrpl37 and Mrpl34 immunostains in spinal sections *of Tg(mnx1:gal4)/(UAS:RFP)* 50 hpf zebrafish. (**C**) Representative confocal images of C9orf72, Mrpl12 and GFP (poly(GP)) co-immunostains in spinal MN from transversal sections of *Tg(mnx1:gal4)/(UAS:RFP)* 50 hpf mismatch + poly(GP) zebrafish. Scale bars: 10 µm. * p<0.05; *** p<0.001. Data are presented as mean + sem.

**
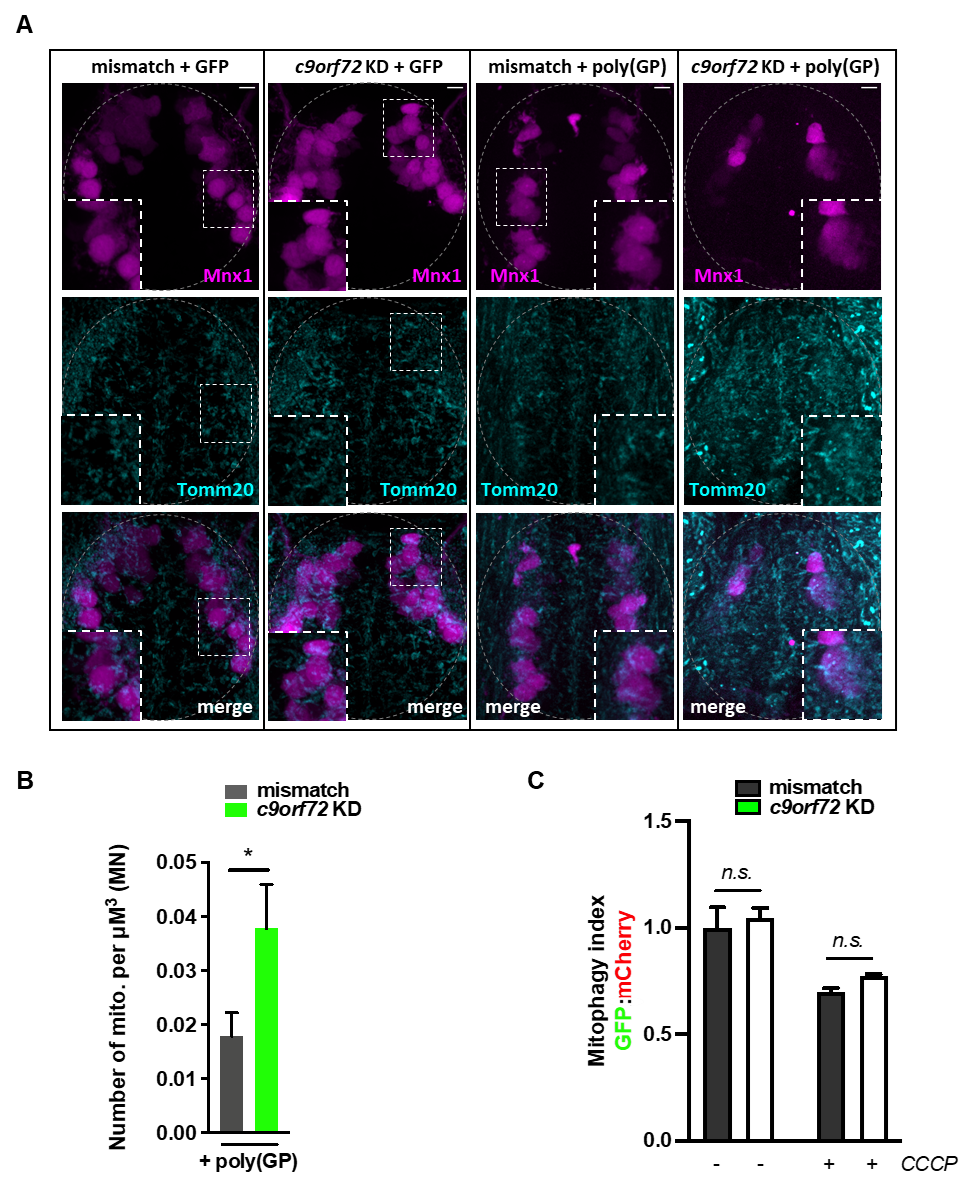
**

**Figure S6.** Mitophagy is altered in *c9orf72* KD + poly(GP) zebrafish spinal motor neurons (MN). (**A**) Representative images from Tomm20 immunolabeling in spinal sections of *Tg(mnx1:gal4)/(UAS:RFP)* 50 hpf zebrafish, showing a disturbed mitochondria signal pattern in spinal MN form *c9orf72* KD + poly(GP) conditions. The dot lines frame representative motor neurons. Zoomed-in views are provided in the inserts. Scale bars: 5 µm. (**B**) Number of mitochondria per MN volume showing an increase in *c9orf72* KD + poly(GP) condition. (**C**) Quantification of mitophagy index of 50 hpf zebrafish showing no difference in mitophagy activity between mismatch and *c9orf72* KD conditions (without poly(GP)). Dissociated cells were treated, or not, with carbonyl cyanide 3-chlorophenylhydrazone (CCCP), as a positive control of mitophagy induction. *<p0.05; *n.s.* non significant. Data are presented as mean + sem.


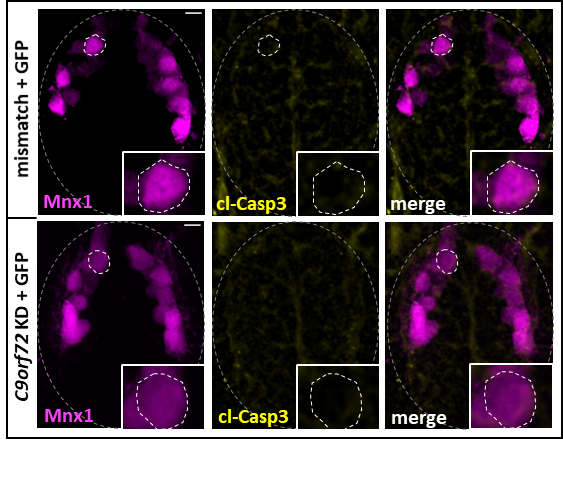


**Figure S7**. Casp3 is not cleaved upon *c9orf72* KD in the absence of poly(GP). Representative images from cleaved Casp3 (cl-Casp3) immunolabeling in spinal sections of *Tg(mnx1:gal4)/(UAS:RFP)* 50 hpf zebrafish, showing no difference in cl-Casp3 fluorescence intensity between motor neurons from these conditions. The dot lines encircle a representative motor neuron. A zoomed-in view is provided in the insert. Scale bar: 5 µm
