# Supplementary material for: Poly-GP accumulation due to C9orf72 loss of function induces motor neuron apoptosis through autophagy and mitophagy defects
Source: Autophagy. 2024 Sep 24;20(10):2164–85. doi: 10.1080/15548627.2024.2358736 (PMC11423671; doi:10.1080/15548627.2024.2358736)

Immunoblot of Fig. 2B

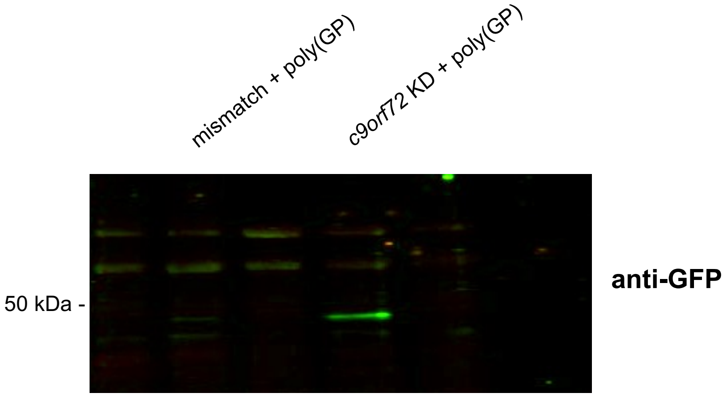

Immunoblot Fig. 2D

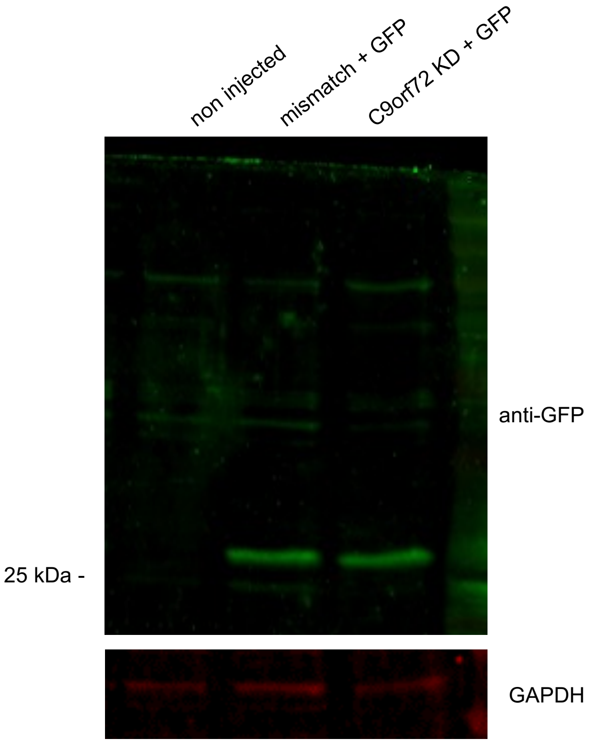

Immunoblot of Fig. 3D

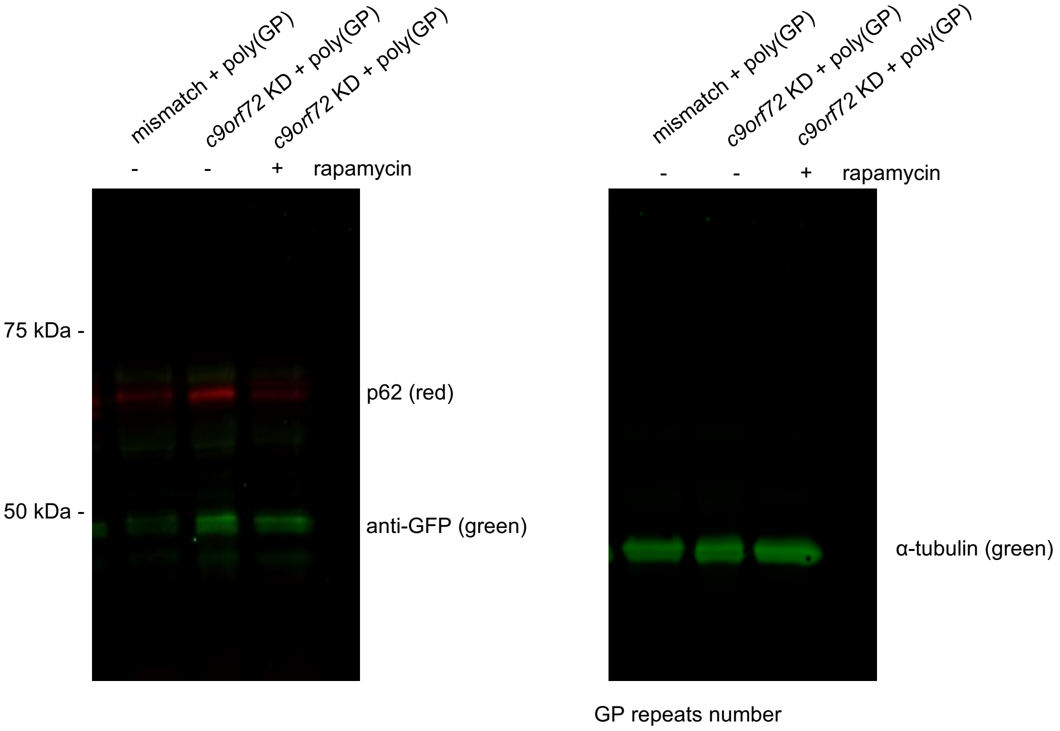

Immunoblot of Supplementary Fig 2A

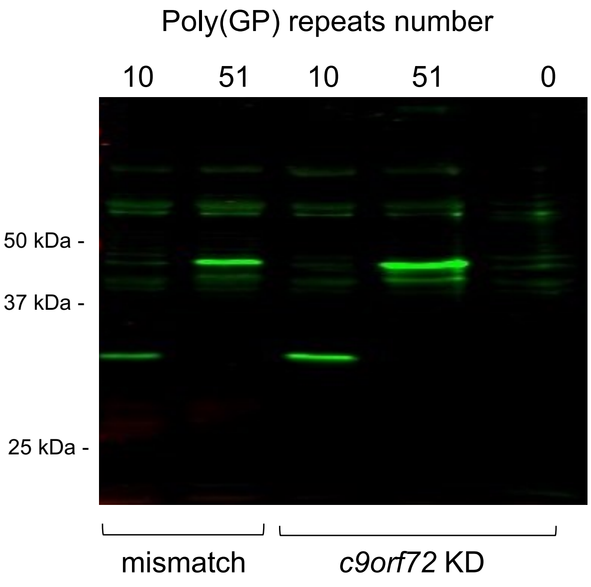

C9-RANT

Immunoblot of Supplementary Fig. 2C

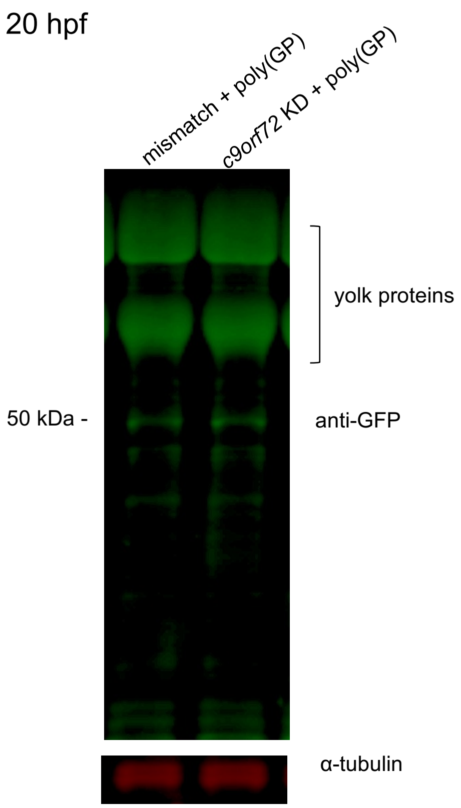

Immunoblot of Supplementary Fig. 2E

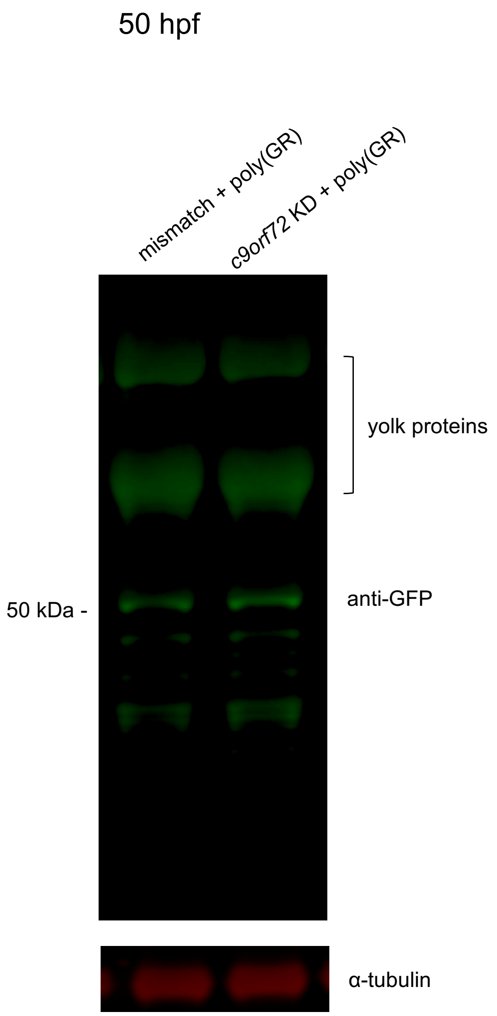

Immunoblot of Supplementary Fig. 3B

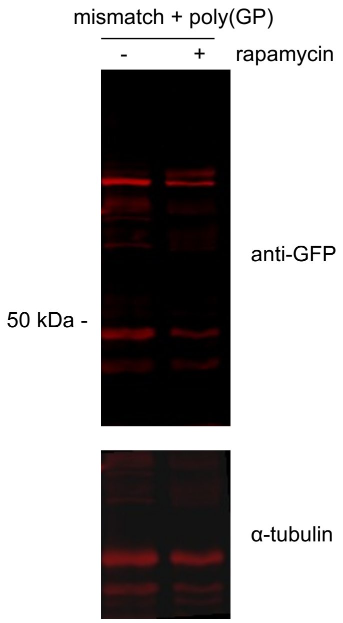

Supplement: Supplemental Material [file KAUP_A_2358736_SM0989.zip › suppl/Source immunoblots.pdf]
